# MicroRNA let-7f-5p regulates PI3K/AKT/COX2 signaling pathway in bacteria-induced pulmonary fibrosis via targeting of *PIK3CA* in forest musk deer

**DOI:** 10.7717/peerj.14097

**Published:** 2022-10-05

**Authors:** Wei Zhao, Jianguo Cheng, Yan Luo, Wenlong Fu, Lei Zhou, Xiang Wang, Yin Wang, Zexiao Yang, Xueping Yao, Meishen Ren, Zhijun Zhong, Xi Wu, Ziwei Ren, Yimeng Li

**Affiliations:** 1 College of Veterinary Medicine, Sichuan Agricultural University, Wenjiang, Sichuan Province, China; 2 Sichuan Institute of Musk Deer Breeding, Dujiangyan, Sichuan Province, China

**Keywords:** Bacteria-induced pulmonary fibrosis, Forest musk deer, Let-7f-5p, PIK3CA gene, PI3K/AKT/COX2 signaling pathway

## Abstract

**Background:**

Recent studies have characterized that microRNA (miRNA) is a suitable candidate for the study of bleomycin/LPS-induced pulmonary fibrosis, but the knowledge on miRNA in bacteria-induced pulmonary fibrosis (BIPF) is limited. Forest musk deer (*Moschus berezovskii*, FMD) is an important endangered species that has been seriously affected by BIPF. We sought to determine whether miRNA exist that modulates the pathogenesis of BIPF in FMD.

**Methods:**

High-throughput sequencing and RT-qPCR were used to determine the differentially expressed miRNAs (DEmiRNAs) in the blood of BIPF FMD. The DEmiRNAs were further detected in the blood and lung of BIPF model rat by RT-qPCR, and the targeting relationship between candidate miRNA and its potential target gene was verified by dual-luciferase reporter activity assay. Furthermore, the function of the candidate miRNA was verified in the FMD lung fibroblast cells (FMD-C1).

**Results:**

Here we found that five dead FMD were suffered from BIPF, and six circulating miRNAs (miR-30g, let-7f-5p, miR-27-3p, miR-25-3p, miR-9-5p and miR-652) were differentially expressed in the blood of the BIPF FMD. Of these, let-7f-5p showed reproducibly lower level in the blood and lung of the BIPF model rat, and the expression levels of PI3K/AKT/COX2 signaling pathway genes (*PIK3CA*, *PDK1*, *Akt1*, *IKBKA*, *NF-*κ*B1* and *COX2*) were increased in the lung of BIPF model rats, suggesting that there is a potential correlation between BIPF and the PI3K/AKT/COX2 signaling pathway. Notably, using bioinformatic prediction and experimental verification, we demonstrated that let-7f-5p is conserved across mammals, and the seed sequence of let-7f-5p displays perfect complementarity with the 3’ UTR of *PIK3CA* gene and the expression of the *PIK3CA* gene was regulated by let-7f-5p. In order to determine the regulatory relationship between let-7f-5p and the PI3K/AKT/COX2 signaling pathway in FMD, we successfully cultured FMD-C1, and found that let-7f-5p could act as a negative regulator for the PI3K/Akt/COX2 signaling pathway in FMD-C1. Collectively, this study not only provided a study strategy for non-invasive research in pulmonary disease in rare animals, but also laid a foundation for further research in BIPF.

## Introduction

Forest musk deer (FMD) is listed as endangered by the International Union of the Conservation of Nature. The adult male FMD could secrete musk, which play an important economic value in traditional Asian medicine and international perfume industries. The population of captive FMD has been hampered by bacterial pneumonia, which accounts for 50% of all deaths. Specifically, pulmonary fibrosis has been presented in almost all of the bacterial pneumonia FMD (Zhang, Wang & Lin, 1997; Zhao et al., 2020). Pulmonary fibrosis is one of the respiratory refractory diseases affecting human and animal health (Williams, 2014), which is a progressive fatal disease accompanied by the inflammatory response, excessive proliferation of fibroblast cell, and deposition of extracellular matrix (ECM) (Williams, 2014; Hsu et al., 2017; Wang et al., 2021a). Many reasons can contribute to the shaping of pulmonary fibrosis, including virus infection, aberrant wound healing, irradiation, and environmental agents (Chioma & Drake, 2017). Of note, accumulating evidence suggests that infectious bacterial, such as *Pseudomonas aeruginosa*, *Streptococcus pneumoniae*, and *Staphylococcus aureus*, also play a role in pulmonary fibrosis (Chioma & Drake, 2017). The earliest evidence of bacterial involvement in pulmonary fibrosis showed that *Mycobacteria* can induce pulmonary lesions by the activator of T lymphocytes (Seggev, Goren & Kirkpatrick, 1984). By using the pulmonary fibrosis model, Knippenberg et al. (2015) uncover a novel mechanism that *Streptococcus pneumoniae* causes progression of established pulmonary fibrosis in mice through releasing pneumolysin. In addition, the correlation between lung microbiome and pulmonary fibrosis was analyzed by 454 pyrosequencing, and the results showed that the pulmonary fibrosis progression is associated with the presence of *Streptococcus* genera (Han et al., 2014). Currently, the mechanism of bleomycin/LPS-mediated pulmonary fibrosis has been widely studied in recent years, much less data is available in BIPF (Chioma & Drake, 2017).

MiRNAs are non-coding small RNAs that play vital roles in the posttranscriptional regulation of gene expression (Chen et al., 2008). In recent years, studies have shown that miRNAs have been widely used in the study of the pathogenic mechanisms of many diseases, including infectious diseases (Bhaskaran & Mohan, 2014). Most importantly, Chen et al. (2008) reported that a barrage of miRNAs are present in body fluids (blood, saliva, tears, urine, milk and seminal fluid), termed circulating miRNAs, which might play a role in information transfer from organs/tissue to the body fluid. Thus, the specific expression profiles of circulating miRNAs can reflect changes in expression in organs/tissue. Slota et al. (2019) study in Père David’s deer chronic wasting disease found that the expression profile of circulating miRNAs were changed in prion disease individual, of note, miR-148a-3p, miR-186-5p, and miR-30e−3p were proved to have a strong correlation with prion disease. The follow-up studies have characterized that circulating miRNAs are suitable candidates to serve as a study strategy for pulmonary fibrosis (Liu et al., 2021). Huang et al. (2012) reported that miR-125b-5p, miR-128, miR-30e, and miR-20b were significantly changed in lung tissue and in plasma of fibrosis mice. Chouri et al. (2018) demonstrated that circulating miR-483-5p could regulate the expression of fibrosis-related genes in fibroblast and endothelial cells by screening and functional study of miRNA in mice serum.

At present, the mechanism of bleomycin/LPS-mediated pulmonary fibrosis has been widely studied in recent years, but the pathogenesis of BIPF is limited (Liu et al., 2018; Chioma & Drake, 2017). Herein, we sought to determine whether miRNAs exist that modulates the pathogenesis of BIPF in FMD. Using high-throughput sequencing and experimental verification, we have confirmed that circulating let-7f-5p is differentially expressed in blood of BIPF FMD and rats compared to the healthy control. Furthermore, the role of let-7f-5p in regulating the PI3K/AKT/COX2 signaling pathway was validated in the lung of BIPF-infected rat and FMD-C1, which implied that let-7f-5p could regulate the PI3K/AKT/COX2 signaling pathway in BIPF. Collectively, our results uncover a novel role for let-7f-5p in BIPF, which provided promising knowledge for further BIPF researches.

## Materials & Methods

### FMD experiment

#### Sample collection

Blood samples were collected from five healthy (H1-H5; three male and two female, *n* = 5) and five dead (P1-P5; three male and two female, *n* = 5) FMD (Sichuan Institute of Musk Deer Breeding) and stored at −80 °C in the blood RNA Shield tube (Tianmo, Beijing, China). The sick FMD were looked after by us throughout the day. When the condition worsened, we monitored their breathing, heartbeat, pulse, and reflexes. With the disappearance of the above indicators, we determined that the FMD died, and performed an autopsy on the FMD, it was observed that the lungs were surrounded by peptone-like exudate. To meet the required standards of blood sample for testing, the cause of death of FMD was detected by pathogen detection and histopathological observation, molecular identification based on 16S rDNA sequences was conducted using general primers 27F and 1492R as previously described (Zhao et al., 2017). Besides, to examine the degree of fibrosis, the lung specimens were harvested and fixed with 4% paraformaldehyde solution for histological analysis with Masson’s trichrome and Picrosirius red staining. The animal study protocol was approved by the Ethics Committee of Sichuan Agricultural University (protocol code No. SYXK2019-187).

#### RNA extraction and RNA library construction

The RNA was extracted from six mL blood from each individual healthy (*n* = 5) and dead (*n* = 5) FMD using a Total RNA extraction kit (Tianmo, Beijing, China) following the manufacturer’s instructions. The statistical power of this experimental design, calculated in G*Power software (3.1.9.7, http://www.gpower.hhu.de/) (Kang, 2021) is 0.42. The integrity of RNA was assessed using electrophoresis with 1.0% denatured agarose gel and Agilent 2100 Bioanalyzer (Agilent Technologies, Santa Clara, CA, USA). The RNA concentration was determined by a NanoDrop ND1000 system (Thermo Fisher Scientific, Waltham, MA, USA). The RNA meeting the following requirements was used for library construction: (a) The total RNA concentration is greater than 200 ng/µL; (b) RNA integrity number ≥ 8; (c) 28S/18S ratio ≥ 1.8. The small RNA libraries were generated using NEBNext® Multiplex Small RNA Library Preparation kit (New England Biolabs, Ipswich, MA, USA) according to the manufacturer’s instructions. The RNA was reverse transcribed into cDNA immediately using Super Script II Reverse Transcriptase (Invitrogen, Waltham, MA, USA). Fragmented libraries were enriched by PCR amplification, and added with universal PCR primers and an index (X) primer. Prior to sequencing, the quality and quantity RNA libraries were evaluated by Agilent 2100 Bioanalyzer with Agilent High Sensitivity DNA Kit and Quant-iT PicoGreen dsDNA Assay Kit, respectively. Finally, qualified libraries were sequenced on Illumina NextSeq 500 sequencing platform producing 75-bp single end reads (>23 million for each sample) at Personal Biotechnology Co., Ltd (Shanghai, China).

#### RNA sequencing data analysis

After getting the raw data (5 healthy and 5 dead FMD blood samples), the ligated adapter sequences were removed by Cutadapt software (https://cutadapt.readthedocs.io/), the sequence data of low quality reads (average quality score <20; read lengths <18 and >32 nt) were discarded (Sun et al., 2019a). Next, the Rfam database (http://rfam.janelia.org/) was used to extract the reads of rRNAs, tRNAs, snRNAs, and snoRNAs. The rest of reads were aligned to the miRBase 21 databases (http://www.mirbase.org/), and achieved the conservative miRNA (each miRNA was allowed ≤ 2 base mismatches). The expression level of the known miRNA was quantified as Transcripts Per Million values. The DESeq2 package (http://www.bioconductor.org/packages/release/bioc/html/ DESeq2.html) was used to analyze DEmiRNAs with *p* < 0.05 and —log_2_fold change— >1. Subsequently, miRanda v3.3a (http://cbio.mskcc.org/microrna_data/manual.html) and TargetScan 7.2 (http://www.targetscan.org/vert_72/) were used to predict the target genes of DEmiRNAs. DEmiRNAs-target gene network was visualized using the Cytoscape software (ver. 3.6.1; http://www.cytoscape.org). Functions and metabolic pathways of these target genes were classified by the Kyoto Encyclopedia of Genes and Genomes (KEGG) pathways, and KEGG pathways were screened at *p*-value <0.05 and false discovery rate (FDR) <0.05.

#### RT-qPCR quantification of the DEmiRNAs in FMD blood

In this study, 1.5 µL of 20 pmol/µL synthetic *C. elegans* miRNA (*cel*-miR-39: 5′-UCACCGGGUGUAAAUCAGCUUG-3′) was added to the blood, which was used for sample-to-sample normalization (Kroh et al., 2010; Hromadnikova et al., 2017). Then, the total RNA of each individual healthy (*n* = 5) and dead (*n* = 4) (All the blood sample of a dead FMD (P4) was used to miRNA-seq) FMD blood was extracted using Total RNA extraction kit (Tianmo, Beijing, China) following the manufacturer’s instructions. The RNA was reverse transcribed into miRNA cDNA immediately using miRcute Plus miRNA First-Strand cDNA synthesis Kit (TianGen, Beijing, China). The DEmiRNAs were detected using miRcute Plus miRNA qPCR Kit (SYBR Green) (TianGen, Beijing, China). The PCR conditions were as follows: initial denaturation at 95 °C for 15 min, 5 thermal cycles (94 °C for 20 s, 64 °C for 30 s, 72 °C for 34 s) to enrichment of target miRNAs, 45 cycles of 94 °C for 20 s and 60 °C for 34 s. The DEmiRNAs forward primers were designed based on mature miRNA sequences and reverse primers were provided by the miRcute Plus miRNA qPCR Kit (SYBR Green) (TianGen, Beijing, China). The target miRNAs in the blood were normalized using the synthetic *C. elegans cel*-miR-39 (Kroh et al., 2010). The sequences of the DEmiRNAs primers used are listed in Table S1. The relative DEmiRNAs expression levels were calculated using the 2^−ΔΔCT^ method.

### Rat experiment

#### Construction of BIPF model and sample collection

In order to establish BIPF model, we carried out a pilot study and found that the pathogenic bacteria isolated from FMD lung, including *Klebsiella pneumoniae* strain DJY-1, *Pseudomonas aeruginosa* strain YW1 and *Streptococcus equinus* strain FMD1, which could cause bacterial pneumonia in rodents (Zhao et al., 2021). A total of 20 adult female Wistar rats (8-week-old; specific-pathogen-free) (Chengdu Dossy Experimental Animals Co, Ltd, Chengdu, 610000, China) were randomly divided into four groups, which received sterile physiologic saline as control group (CG group, *n* = 5), *Klebsiella pneumoniae* (KG group, *n* = 5), *Pseudomonas aeruginosa* (PG group, *n* = 5) and *Streptococcus equinus* (SG group, *n* = 5) by nasal drip under anesthesia as described in Chen et al. (2017). For group KG, PG and SG, the three pathogens were administered at a dose of 10^8^∼10^9^ CFU per rat. In order to prevent cross-contamination of pathogens, each group rats were housed in a separate cage with enough distance. The rats had free access to food and water *ad libitum* with a 12 h light/dark cycle. On day 21, the rats were weighed and sacrificed after anesthesia, and the blood was collected into blood RNA Shield tube (Tianmo, Beijing, China). The lung samples were weighed and collected, and then one part of the lung tissue was collected into RNALater™ RNA Stabilization Reagent (Beyotime, Shanghai, China). The blood and lung specimens were stored at liquid nitrogen until total RNA extraction. Immediately afterward, the other part was fixed with 4% paraformaldehyde solution and stained with hematoxylin-eosin (HE), masson’s trichrome and picrosirius red staining.

#### RT-qPCR quantification of the DEmiRNAs in rat blood and lung

The method of DEmiRNAs in rat blood and lung determination was the same as above. There is a slight difference in the lung miRNA detection that the target miRNAs in the lung were normalized using U6 snRNA (Wang et al., 2017). The sequences of the DEmiRNAs primers used are listed in Table S1. The relative DEmiRNAs expression levels were calculated using the 2^−ΔΔCT^ method.

#### RT-qPCR analysis of the BIPF-related genes in rat lung

We evaluate the expression level of pulmonary fibrosis-related genes, including transforming growth factor-*β*1 (*TGF-β1*), tumor necrosis factor- *α* (*TNF-α*), and PI3K/AKT/COX2 signaling pathway genes: phosphatidylinositol-4,5-bisphosphate 3-kinase catalytic subunit alpha (*PIK3CA*), phosphoinositide-dependent protein kinase 1 (*PDK1*), serine/threonine kinase 1 (*Akt1*), inhibitor of nuclear factor kappa-B kinase subunit alpha (*IKBKA*), nuclear factor kappa B subunit 1 (*NF-κB1*) and cyclooxygenase-2 (*COX2*), in the rat lung tissue using RT-qPCR analysis. The total RNA was extracted from the rat lung using Total RNA extraction kit (Tianmo, Beijing, China), and reversed transcribed using HiScript III 1st Strand cDNA Synthesis Kit (+gDNA wiper) (Vazyme Biotech Co., Ltd, Nanjing, China). The *β*-actin was used as reference gene. The sequences of the gene primers used are listed in Table S2. The relative gene expression level was calculated using the 2^−ΔΔCT^ method.

#### Western blot analysis

Total protein was extracted from rat lung tissue using Tissue or Cell Total Protein Extraction Kit (Sangon Biotech Co., Ltd, Shanghai, China) and protein concentration was determined by bicinchonininc acid (BCA) Protein Assay Kit (TianGen, Beijing, China). The PI3K/AKT/COX2 signaling pathway related proteins (PI3K, AKT1, and COX2) were analyzed by western blot. Equal amounts of 50 µg total proteins were separated on 10% SDS-PAGE gels and then transferred onto polyvinylidene fluoride (PVDF) blotting membranes. After being blocked with 5% bovine serum albumin (BSA) in phosphate buffer, the membranes were incubated with primary antibodies at 4 °C for the night. The primary antibodies used were PIK3CA rabbit polyclonal antibody (diluted 1:2,000), phospho-AKT1 (Ser473) rabbit polyclonal antibody (diluted 1:800), anti-COX2 antibody (diluted 1:1,000), and *β*-Actin rabbit monoclonal antibody (diluted 1:1,000). Following incubation with goat anti-rabbit secondary antibody (diluted 1:1,000) for 2 h at room temperature, the protein bands were visualized using an enhanced chemiluminescence detection system. The *β*-actin was used as an internal control for assessing equal loading of total protein among wells. In this study, the primary and secondary antibodies were purchased from Beyotime biological technology Co., Ltd (China) and Boster biological technology Co., Ltd (America).

### Generation of Luciferase Constructs and Luciferase Assays

The *PIK3CA* was predicted as target gene of let-7f-5p. For experimental validation of the *PIK3CA* 3′ UTR (untranslated region) as a target of let-7f-5p, the luciferase reporter plasmid was constructed, and a 200-bp fragment of the *PIK3CA* mRNA 3′ UTR containing the predicted let-7f-5p binding site (seed sequence) (*PIK3CA* mRNA 3′ UTR-WT) and its mutant sequence (*PIK3CA* mRNA 3′ UTR-MUT) were synthesized by Sangon and cloned into psiCHECKTM-2 vector (Sangon Biotech Co., Ltd., Shanghai, China) based on the *Xho* I and *Not* I restriction sites. The recombinant plasmids were confirmed by double digest and PCR amplification, the primers used are listed in Table S3. The FAM-labeled let-7f-5p mimics (5′-UGAGGUAGUAGAUUGUGUAGU-3′), let-7f-5p inhibitor (5′-ACUAUACAAUCUACUAC CUCA-3′) and mimics NC (5′-UUGUACUACACAAAAGUACUG-3′) were synthesized by Sangon. For transient transfections, the optimum concentration of let-7f-5p mimics, let-7f-5p inhibitor and mimics NC were carried out in HEK293T cells with the luciferase reporter constructs using Lipo8000™ Transfection Reagent (Beyotime Biotechnology, Co., Ltd., Shanghai, China). After 24 h of transfection, HEK293T cells were lysed by TransDetect^®^ Double-Luciferase Reporter Assay Kit (TransGen Biotech Co., Ltd. Beijing, China) and the luciferase activity was measured by a Full wavelength scanning, multifunction reading instrument (Thermofisher, USA). The Renilla luciferase activities were normalized by that of firefly luciferase.

### The correlation analysis between let-7f-5p and PI3K/AKT/COX2 signaling pathway in FMD fibroblast cells

#### Package of the recombinant adeno-associated virus (rAAV)

The pAAV-CMV-eGFP and helper plasmids were obtained from Prof Zhao, Huazhong Agricultural University, China (Huang et al., 2021). The FMD pri-let-7f-5p sequence (SPDX01004329: 114203-114718) was cloned into pAAV-CMV-eGFP plasmids based on the Hind restriction sites and homologous arm. The recombinant plasmids (rpAAV-pri-let-7f-5p) were confirmed by double digest and PCR amplification. Primers were designed based on the pAAV-CMV-eGFP sequence information (F: TGCTGCCCGACAACCA; R: CCCTTGCTCCATACC AC). Then, a packaging approach, which have been previously reported (Huang et al., 2021), was used to produce the rAAV, and the AAV was set as the blank control. Subsequently, the rAAV and AAV copy number was assessed by RT-qPCR and transduction assay according to the methods of Meng, Zhang & Zhang (2013). Finally, we successfully packaged the rAAV virus, which can be applied for let-7f-5p overexpressing in FMD-C1 cells (Fig. S1).

#### Effects of let-7f-5p on PI3K/AKT/COX2 signaling pathways

To provide further evidence that the PI3K/AKT/COX2 signaling pathway was regulated by let-7f-5p in FMD lung, we successfully isolated and cultured primary FMD-C1 cell, a detailed procedure of the methods of cell separation can be found in a previous study (Liu et al., 2012). FMD-C1 cells were transfected with let-7f-5p mimics and mimics NC using Lipo8000™ Transfection Reagent. Subsequently, FMD-C1 cells were infected with rAAVs and AAVs at a multiplicity of infection of 200 and incubated at 37 °C for 8 days. Finally, the expression level of PI3K/AKT/COX2 signaling pathway genes (*PIK3CA*, *PDK1*, *Akt1*, *IKBKA*, *NF-κB1* and *COX2*) were detected by RT-qPCR. The total RNA was extracted from the rat lung and FMD-C1 using Total RNA extraction kit (Tianmo, Beijing, China), which was reversed transcribed using HiScript III 1st Strand cDNA Synthesis Kit (+gDNA wiper) (Vazyme Biotech Co., Ltd, Nanjing, China), the *β*-actin was used as reference gene. The sequences of the gene primers used in this study are listed in Table S2. The relative gene expression level was calculated using the 2^−ΔΔCT^ method.

### Statistical analysis

Statistical analyses were performed using GraphPad Prism 7.00 software. Data have been expressed as means ± Standard Error of Mean. Two-tailed student’s t test was performed to test for differences between the two groups, *p-value <0.05; **p-value <0.01.

## Results

### Pathogen identification and pathology analysis

**Figure 1:**
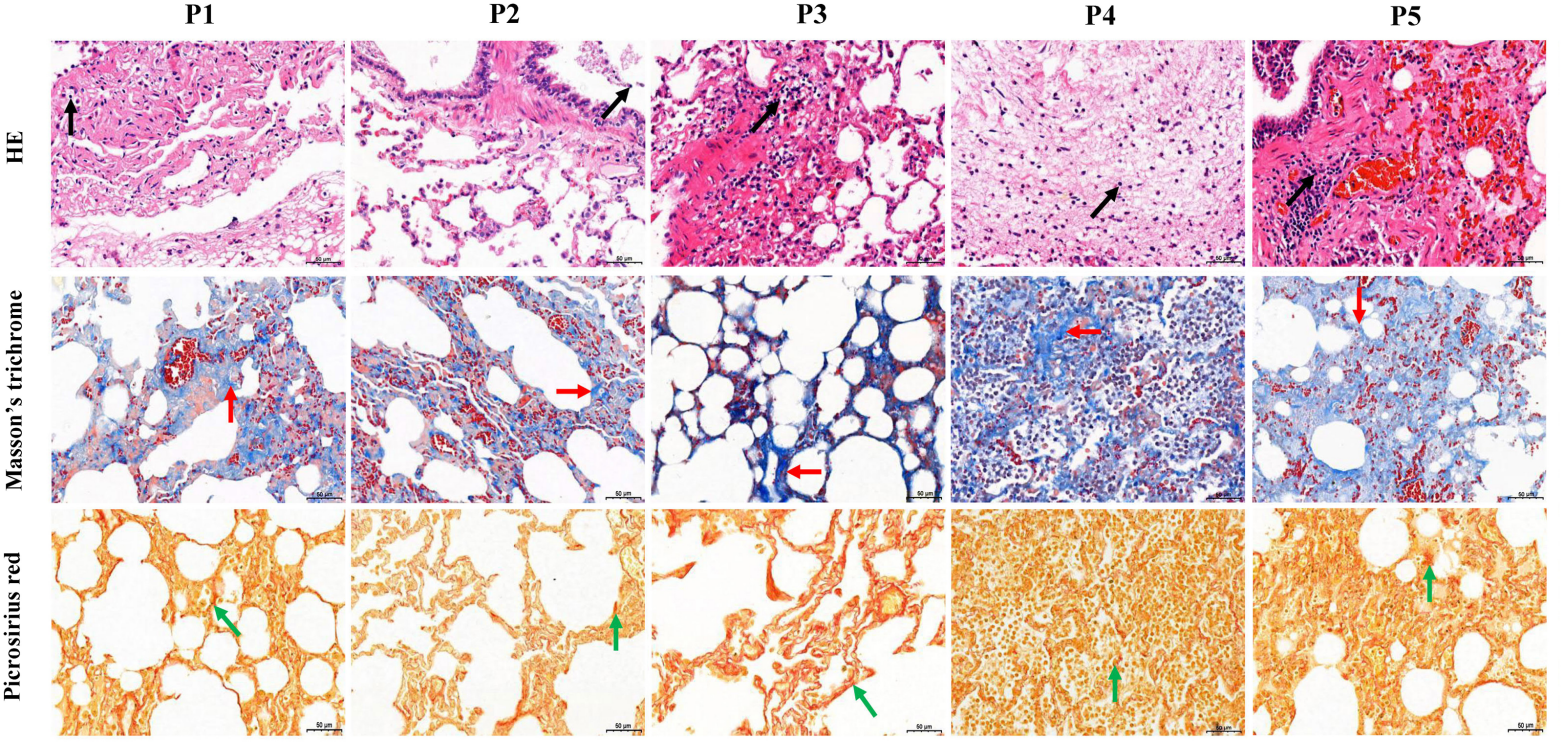
Histological analysis of five dead forest musk deer lung tissue (400×). The black, red and green arrow indicates the lymphocytes, fibrin and collagen, respectively; P1-P5 indicates the five dead forest musk deer, respectively.

The five FMD individuals died of bacterial pneumonia, and a pathogen was isolated from the lung of each of the dead FMD, namely *Pseudomonas aeruginosa* strain YW1 (Genbank NO. MN027911.1), *Pseudomonas aeruginosa* strain FMDP002 (Genbank NO. MN904450.1), *Klebsiella pneumoniae* strain DJY-1 (Genbank NO. OK036428), *Streptococcus equinus* strain FMD1 (Genbank NO. MK652875.1) and *Trueperella pyogenes* strain ZW1 (Genbank NO. MN027909.1), respectively (Fig. S2). In the five FMD lungs, histopathological examination showed some similar pathological changes in each individual, mainly including the normal structure of alveolus was lost, fibrous tissue was increased, and the lung interstitium was filled with a large number of fibroblast cells, neutrophils and lymphocytes. Picrosirius red and Masson’s trichrome staining showed increased fibrin and collagen in lung tissue of FMD (Fig. 1), implying that the five dead FMD were suffered from BIPF.

### Construction of BIPF model

**Figure 2:**
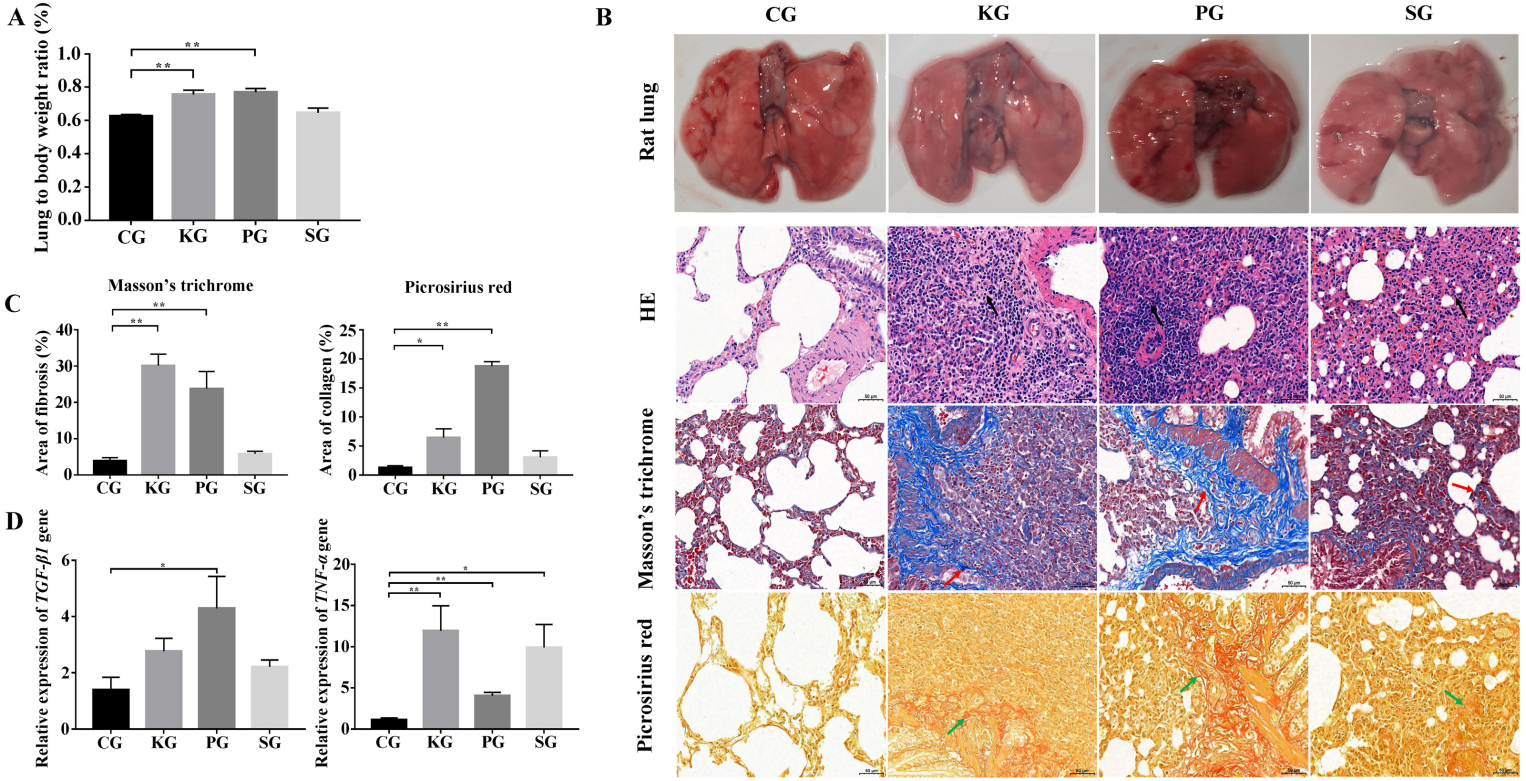
Construction of rat model of bacteria-induced pulmonary fibrosis based forest musk deer pathogens. (A) The ratio of wet lung to body weight of four group rats. (B) Histological analysis of rat lung tissues with HE, Masson’s trichrome and Picrosirius red staining (400×). (C) The quantification of Masson’s trichrome and Picrosirius red staining. (D) The expression level of TGF-*β*1 and TNF- *α* in the lung of rat (*p*-values were calculated using Student’s *t* test. * *p*-value < 0.05, ** *p*-value < 0.01). The black, red and green arrow indicates the lymphocytes, fibrin and collagen, respectively. The “CG”, “KG”, “PG”, and “SG” indicates the control group , *Klebsiella pneumoniae* group, *Pseudomonas aeruginosa* group and *Streptococcus equinus* group, respectively.

In the rat model, the ratio of the wet lung to body weight was calculated at 21 days post infection. Compared with the control group CG, the test group KG and PG exhibited significantly higher wet lung to body weight ratio (*p* < 0.01), but no significant change in test group SG (Fig. 2A). The results of the histological analysis are as follows: in the CG group, the normal structure of the rat lung was complete and clear, and no hyperplasia or thickening of connective tissue was found. The bronchial structure at all levels was complete and clear, and the alveolar epithelial cells were normal in shape without obvious degeneration, necrosis or exfoliation. Additionally, no obvious inflammatory cell infiltration and fibrous hyperplasia were observed in the interstitium. In three test group rats, the alveolar wall was collapsed in the rat lung tissue. The collagen fibers were proliferated in the lung interstitium. Besides, a large amount of fibroblast cells, inflammatory cells (mainly neutrophils and lymphocytes) were infiltrated in the lung interstitium (Fig. 2B). Picrosirius red and Masson’s trichrome staining showed that the lung tissues of three test groups (KG, PG and SG) exhibited severe fibrin and collagen secretion compared with that of the control group (Figs. 2B and 2C). Of note, the expression levels of TGF-*β*1 and TNF-*α* were both upregulated in the lung of test group rats compared with that of the control group (Fig. 2D). These results indicate that the lung of rat in three test groups had developed fibrosis.

### Analysis of BIPF related miRNAs

**Figure 3:**
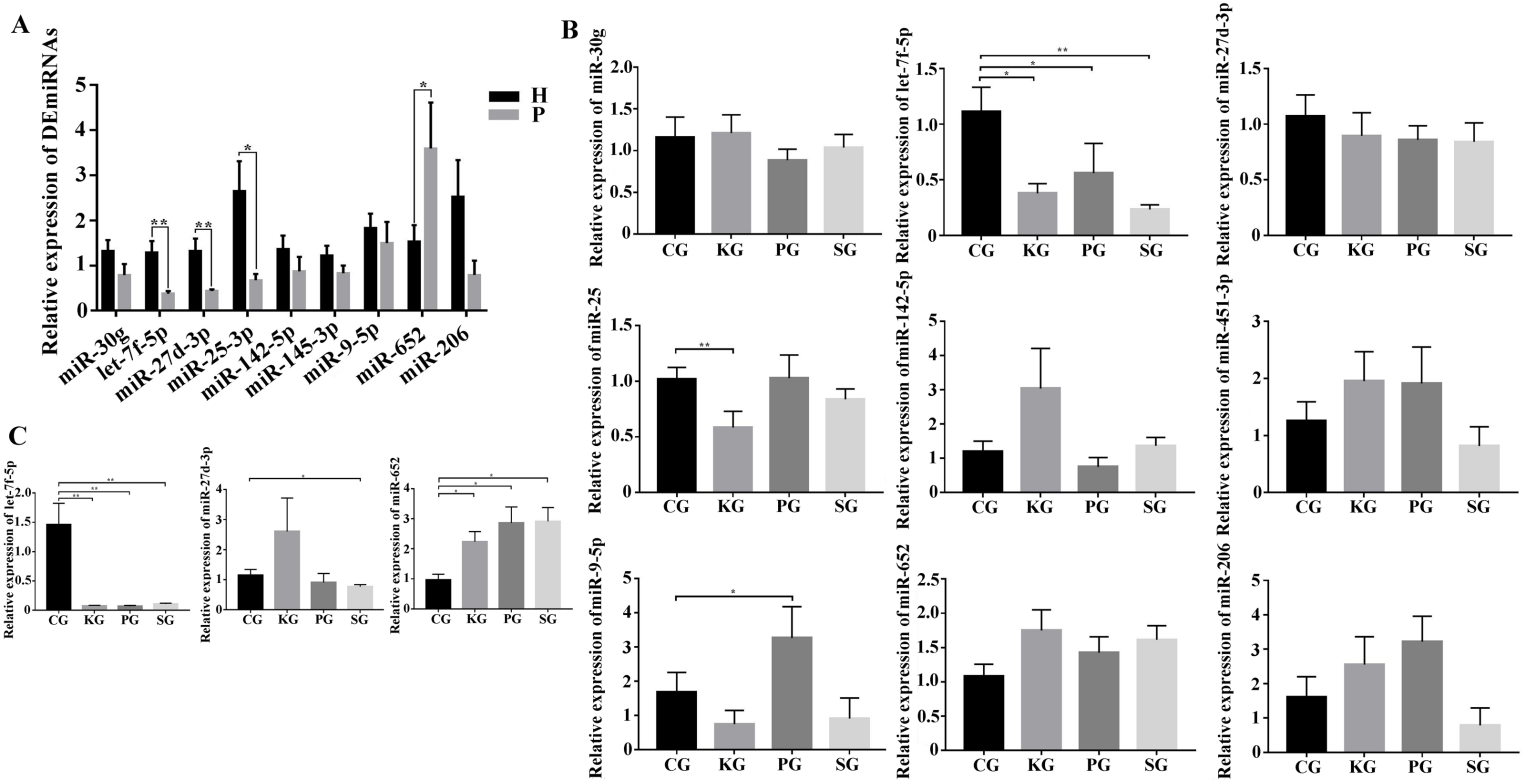
Screening of key miRNAs involved in bacteria-induced pulmonary pulmonary fibrosis. (A) RT-qPCR analysis of differentially expressed miRNAs in blood between healthy and bacteria-induced pulmonary fibrosis forest musk deer (The “H” and “P” indicates the healthy and dead group, respectively). (B) RT-qPCR analysis of differential expression miRNAs in blood between control group and test group rats. (C) RT-qPCR analysis of differential expression miRNAs in lung between control group and test group rats (*p*-values were calculated using Student’s t test. *p -values < 0.05, ** *p*-values < 0.01). The “CG”, “KG”, “PG”, and “SG” indicates the control group, *Klebsiella pneumoniae* group, *Pseudomonas aeruginosa* group and *Streptococcus equinus* group, respectively.

As there is no available miRNA annotation information in miRBase21 for FMD, the known miRNAs were identified by blast searches against all animal reference sequences in miRBase21. Between 25 and 42 million raw reads were obtained for each sample (Table S4). After being trimmed, a total of 313 million clean reads (>18 nt) were obtained. Among these clean reads, small RNA with the length of 18–24 nt was most abundant in FMD blood samples. The miRNAs profiles of each blood samples were identified based on the miRBase21 database after the annotated and unconcerned reads were removed such as rRNA, tRNA, snRNA and snoRNA. A total of 10,088 known miRNAs were identified from 10 blood samples. Among these known miRNAs in the study, nine DEmiRNAs, namely, miR-30g, let-7f-5p, miR-25-3p, miR-27d-3p, miR-451-3p, miR-142-5p, miR-652, miR-9-5p and miR-206-3p, satisfied the screening criteria of *p* < 0.05, —log_2_fold change—>1 between the healthy and dead group FMD. Expression of miR-451-3p, miR-142-5p, miR-652, miR-9-5p and miR-206-3p expression were upregulated in the dead group. Four miRNAs, miR-30g, let-7f-5p, miR-25-3p and miR-27d-3p, were downregulated in the dead group (Table 1). We selected the nine DEmiRNAs to determine their expression patterns using RT-qPCR detection in FMD and rat blood. In the FMD blood, the results of RT-qPCR showed that the trend of 5 DEmiRNAs (miR-30g, let-7f-5p, miR-27-3p, miR-25-3p and miR-652) expression was consistent with the high-throughput sequencing data (Fig. 3A), indicating significant differences in the 6 DEmiRNAs expression between the healthy and dead FMD. In rat model, of the nine miRNAs tested in FMD blood, the let-7f-5p and miR-27d-3p expression level were reduced in the blood of test rat, and the level of miR-652 increased in the three test groups compared with the control group (Fig. 3B). The same changes of let-7f-5p and miR-652 were also observed in the lung of BIPF rats (Fig. 3C).

**Table 1:** The information of differentially expressed miRNAs between healthy and bacteria-induced pulmonary fibrosis forest musk deer.

MiRNAs	Mature sequence	*p*-value	log_2_ fold change	Regulation
miR-30g	UGUAAACAUCCUUCACUCUCAGC	0.020657204	−3.834803637	Down Regulation
let-7f-5p	UGAGGUAGUAGAUUGUGUAGU	0.045518112	−4.008303155	Down Regulation
miR-27d-3p	UUCACAGUGGCUAAGUUCGG	0.040976811	−12.20641173	Down Regulation
miR-25-3p	CAUUGCACUUGUCUCGGC	0.01127513	−3.87762947	Down Regulation
miR-142-5p	CCCAUAAAGUAGAAAGCACUAC	0.043664864	2.442527514	Up Regulation
miR-451-3p	AUGGUAACGGUUCUCUUGCUG	0.02039289	3.609186008	Up Regulation
miR-652	UGAAUGGCGCCACUAGGGUUGUG	0.04590716	^a^	Up Regulation
miR-9-5p	UCUUUGGUUAUCUAGCUGUGUG	0.043801272	^a^	Up Regulation
miR-206	UGGAAUGUAAGGAAGUGUGUGG	0.000474216	^a^	Up Regulation

**Notes.**

a The standardized mean expression of miRNAs in the healthy group is zero by high-throughput sequencing.

### Analysis of BIPF related pathway

**Figure 4:**
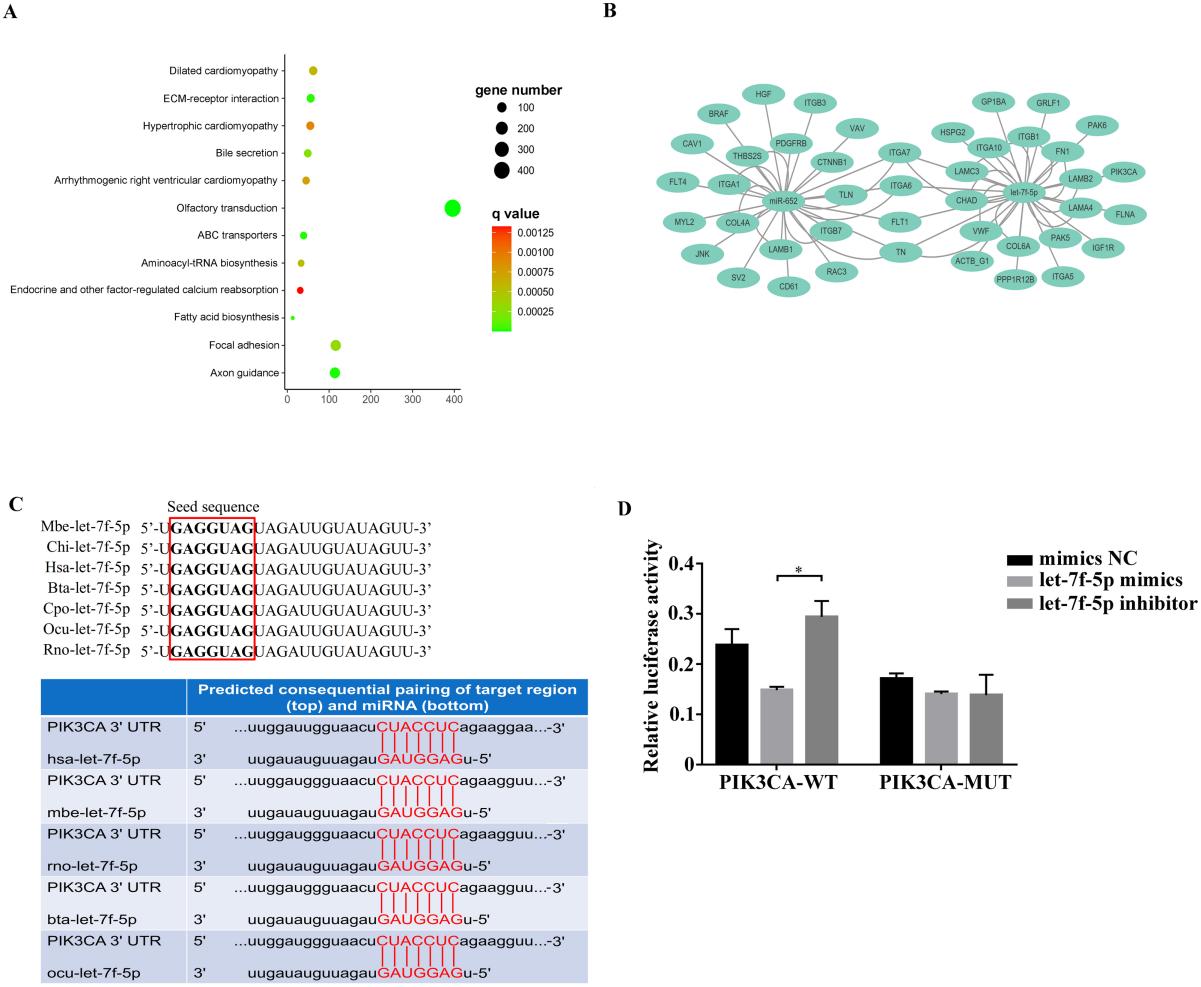
Analysis of the relationship between let-7f-5p and *PIK3CA* gene. (A) KEGG functional enrichment analysis of the target genes of differentially expressed miRNAs. (B) Predicted target genes for let-7f-5p and miR-652 in the ECM-receptor interaction and focal adhesion pathways, respectively. (C) A mature let-7f-5p sequence is conserved among different species, and sequence alignment between let-7f-5p and *PIK3CA* 3′ UTR of forest musk deer, rat, cow, rabbit and human is observed. The sequences in the red frame are seed sequences of let-7f-5p . The “Mbe”, “Chi”, “Hsa”, “Bta”, “Cpo”, “Ocu” and “Rno” indicates the “*Moschus berezovskii*”, “*Capra hircus*”, “*Homo sapiens*”, “*Bos taurus*”, “*Cavia porcellus*”, “*Oryctolagus cuniculus*” and “*Rattus norvegicus*”, respectively . (D) let-7f-5p negatively regulates *PIK3CA* gene. *PIK3CA* mRNA 3′ UTR wild type (*PIK3CA*-WT) and *PIK3CA* mRNA 3′UTR mutant type (*PIK3CA* -MUT) reporter plasmids were cotransfected with the let-7f-5p mimics and let-7f-5p inhibitor in HEK293T cells, and the mimics NC group was also set. The results were expressed as relative luciferase activity (Renilla LUC/Firefly LUC). ( *p*-values were calculated using Student’s t test. * *p*-values < 0.05); “NC” indicates the negative control.

**Figure 5:**
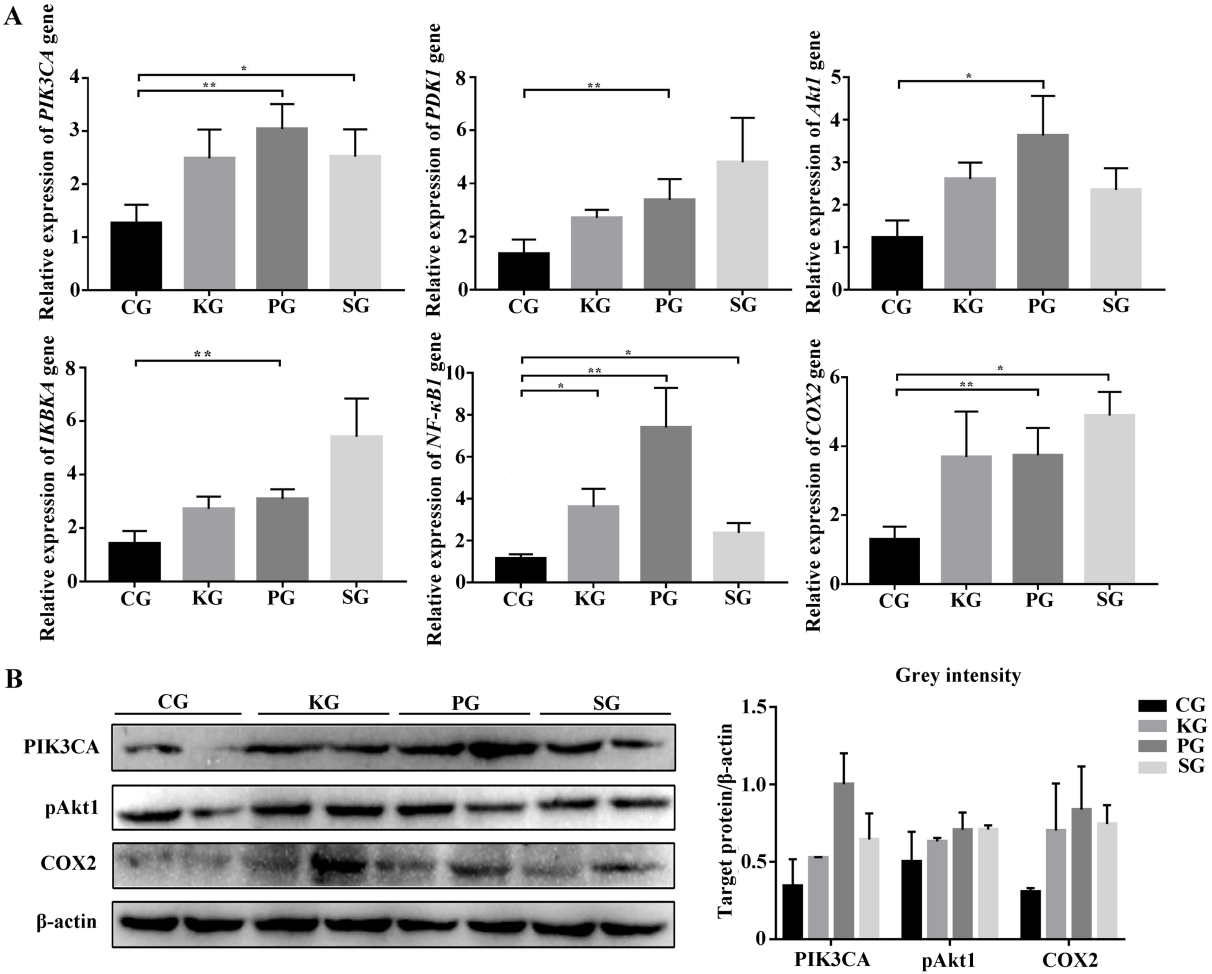
The expression level of PI3K/AKT/COX2 signaling pathway genes in bacteria-induced pulmonary fibrosis rat lung. (A) RT-qPCR analyses of PI3K/AKT/COX2 signaling pathway genes. (B) Western blot for quantification of PI3K/AKT/COX2 signaling pathway related proteins in rat lungs, with *β*-actin as internal control (*p*-values were calculated using Student’s *t* test. *p-values < 0.05, **p-values < 0.01). The “CG”, “KG”, “PG”, and “SG” indicates the control group, *Klebsiella pneumoniae* group, *Pseudomonas aeruginosa* group and *Streptococcus equinus* group, respectively.

Among the KEGG term, twelve KEGG pathways significantly enriched for target genes of the DEmiRNAs (*p* < 0.05; FDR < 0.05) (Table 2, Fig. 4A) based on KEGG analysis. Importantly, the pathway of ECM-receptor interaction and focal adhesion may be of particular importance in this study since these two pathways often associated with lung-related diseases, for example pulmonary fibrosis (Tian et al., 2019). As aforementioned in the target gene analysis, a total of 21 and 39 target genes of the let-7f-5p and miR-652 were involved in ECM-receptor interaction and focal adhesion pathways, respectively (Fig. 4B). It is worth noting that the whole and seed sequences of let-7f-5p are highly conserved across species, and the seed sequence is complementary with the binding site on the *PIK3CA* mRNA (Fig. 4C), suggesting that the let-7f-5p may participate in post-transcriptional regulation of *PIK3CA* gene. The luciferase reporter plasmid was constructed for experimental validation of the relationship between let-7f-5p and *PIK3CA* (Fig. S3). The luciferase reporting assay confirmed the direct target relationship between let-7f-5p and the *PIK3CA* gene (Fig. 4D). In addition, the PI3K/AKT/COX2 signaling pathway genes (*PIK3CA*, *PDK1*, *Akt1*, *IKBKA*, *NF- κB1* and *COX2*) were examined in rat model. Compared to control group, three test groups of rats exhibited increased mRNA expression level of *PIK3CA*, *PDK1*, *IKBKA*, *NF-κB1* and *COX2* genes with significant difference (*p* < 0.05) (Fig. 5A). In addition, the mRNA expression level of *Akt1* gene exhibited a trend of increase in three test group rats, and significantly increased in PG rats (*p* < 0.05) (Fig. 5A). Besides, compared with the control group, three test groups exhibited significantly higher protein expression levels of PI3K, AKT1, and COX2 (Fig. 5B). The high level of the PI3K/AKT/COX2 signaling pathway mRNA and protein expression found in three test group rats, suggesting that there is a potential correlation between BIPF and the PI3K/AKT/COX2 signaling pathway.

**Table 2:** KEGG enrichment analysis of target genes of nine differentially expressed miRNAs.

Rank	KEGG pathways	Target genes	*p*-value	FDR^a^
1	Olfactory transduction	16	2.00445E−08	6.79508E−06
2	Axon guidance	88	7.72678E−06	0.001205408
3	ABC transporters	34	1.06673E−05	0.001205408
4	ECM-receptor interaction	44	1.71932E−05	0.001457125
5	Fatty acid biosynthesis	6	3.11572E−05	0.002112458
6	Bile secretion	38	0.000188414	0.010645395
7	Focal adhesion	91	0.000248674	0.012042906
8	Aminoacyl-tRNA biosynthesis	42	0.000420139	0.017283098
9	Dilated cardiomyopathy	51	0.000458843	0.017283098
10	Arrhythmogenic right ventricular cardiomyopathy	42	0.000566274	0.0191967
11	Hypertrophic cardiomyopathy	46	0.000710711	0.021902822
12	Endocrine and other factor-regulated calcium reabsorption	22	0.001043918	0.02949068

**Notes.**

a FDR indicates the false discovery rate.

### The role of let-7f-5p in the PI3K/AKT/COX2 signaling pathway

**Figure 6:**
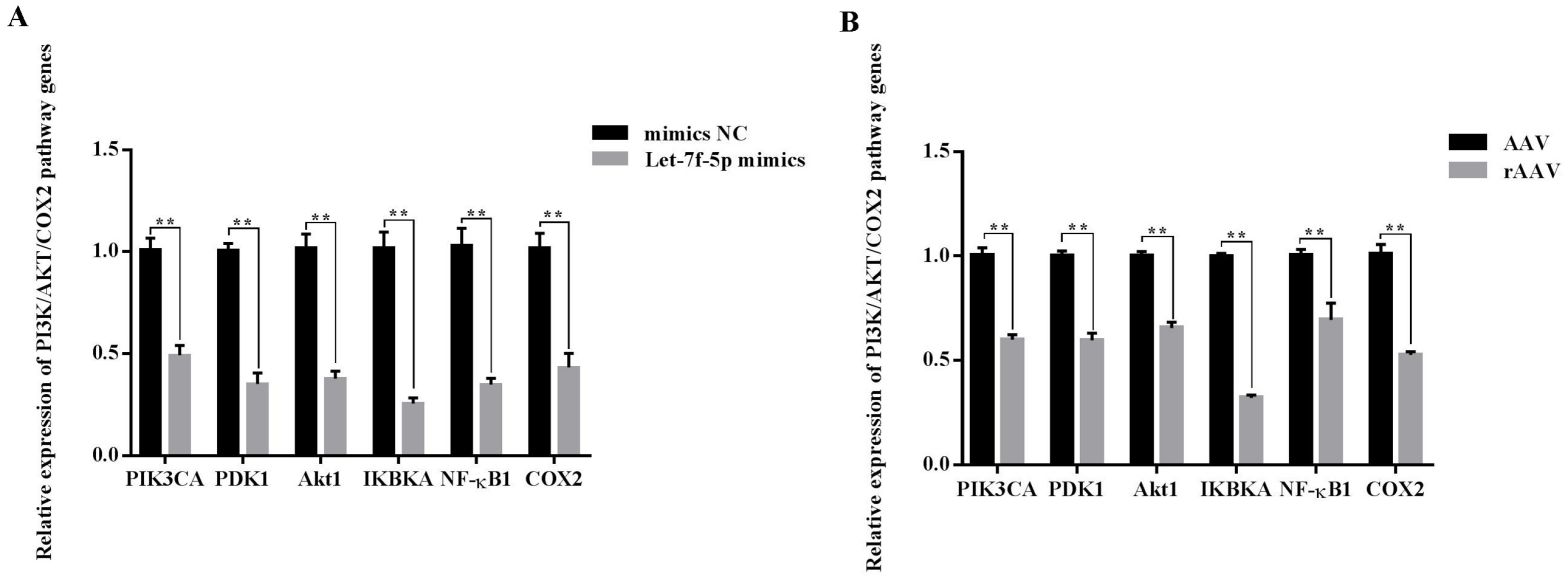
Effect of let-7f-5p on the PI3K/AKT/COX2 signaling pathway in forest musk deer fibroblast cells. “NC” indicates the negative control, “AAV” indicates the adeno- associated virus, “rAAV” indicates the recombinant adeno-associated virus, the *p*-values were calculated using Student’s *t* test. *p-value < 0.05, ** *p*-value < 0.01.

Overall, we hypothesized that the let-7f-5p may participate in the regulation of the PI3K/AKT/COX2 signaling pathway in BIPF FMD. After 24 h of transfection, the *PIK3CA*, *PDK1*, *Akt1*, *IKBKA*, *NF- κB1* and *COX2* expression decreased in let-7f-5p mimics group compared with let-7f-5p mimics NC group (Fig. 6). In addition, the PI3K/AKT/COX2 signaling pathway genes were decreased in rAAV infection FMD-C1 (Fig. 6). These results supported the hypothesis that the let-7f-5p could regulate the PI3K/AKT/COX2 signaling pathway in the FMD lung.

## Discussion

Pulmonary fibrosis is the final stage of many lung diseases in humans, which have also been described in dogs, cats, horses and FMD (Zhang, Wang & Lin, 1997; Zhao et al., 2020; Williams, 2014; Marenzoni et al., 2011). In this study, five FMD were found suffering from BIPF. FMD is an endangered and protected animal in China, just like other protected animals, it is forbidden to conduct animal regression experiments on FMD. However, it is not conducive to the study of BIPF of FMD without healthy control. Therefore, in this study, BIPF model in rat was established by FMD origin pathogens, including *Klebsiella pneumoniae*, *Pseudomonas aeruginosa* and *Streptococcus equinus*. These pathogens are isolated from the FMD lung and have been shown to cause the death of mice (Zhao et al., 2020; Zhao et al., 2021). HE, Picrosirius red and Masson’s trichrome staining showed increased inflammatory cells, fibrin and collagen in the lung of test group rats, demonstrating that these lung tissues are fibrosis. Hsu et al. (2017) have also measured the morphology of pulmonary fibrosis using HE, Picrosirius red and Masson’s trichrome staining. Meanwhile, the level of TGF-*β*1 and TNF-*α* were upregulated in the lung of the test group rats. Similar results have been reported in bleomycin/LPS-induced pulmonary fibrosis model (Hu et al., 2020; Elliot et al., 2019). Reportedly, TGF-*β*1 and TNF-*α* are important cytokines in mediating pulmonary fibrosis and have been demonstrated in many studies (Li et al., 2018). TGF- *β*1 promotes the growth of fibroblast cells and the accumulation of ECM, mainly collagen and fibronectin, and also could inhibit the degradation of ECM (Chioma & Drake, 2017). TNF-*α* has been widely accepted as an important inflammatory cytokine, and has also been reported to exacerbate the inflammatory response and promote TGF-*β*1 secretion in lung fibroblast cells (Wang et al., 2021b). Collectively, these results demonstrated that the origin of FMD pathogen could cause BIPF in rat. Establishment of the rat model of BIPF may lay a foundation for the study of pathogenesis of BIPF in FMD.

In this study, nine DEmiRNAs were found in the blood between the healthy and dead group FMD. It was reported that circulating miRNA may provide information about the disease status of organ/tissue and contain fingerprints for a range of many diseases (Chen et al., 2008), and have been applied to the diagnosis and mechanism research of pulmonary disease in human (Liu et al., 2021). Besides, miRNAs are conserved between species that we can use the same sequence between different animals, and its function could be verified in animal model (Lin, Chang & Ying, 2006). Thus, we used RT-qPCR to analyze the transcript level of the nine FMD blood DEmiRNA in blood and lung of BIPF model rat, and found that let-7f-5p and miR-652 were differentially expressed simultaneously in the rat blood and lung, suggesting that the let-7f-5p and miR-652 might be new candidates for the mechanism study of BIPF. Previous researches in miR-652 analysis have tended to focus on tumor proliferation and metastasis (Sun et al., 2018). The let-7 family miRNAs have previously been shown to participate in regulation of the pulmonary fibrosis. For example, Li et al. (2018) reported that let-7 family miRNA was proved to be an inhibitor that could downregulate estrogen receptor which plays a pivotal role in male-predominant pulmonary fibrosis. Also, Zhu et al. (2011) reported that let-7 plays a significant regulatory role in modulating glucose metabolism via negatively regulating the activity of the insulin-PI3K-mTOR signaling pathway. At the same time, research suggests that the change in glucose metabolism is an important pathogenic process of pulmonary fibrosis, and the effects were linked to the TGF-*β* secretion, ECM synthesis, collagen production, glucose transporter, inflammatory response, and immune response (Selvarajah et al., 2021; Yin et al., 2021; Gopu et al., 2020). These studies reinforce our notion that the let-7 family and PI3K may play important roles in the pulmonary fibrosis. Let-7f-5p, a member of the let-7 family, has been reported to remit pulmonary fibrosis through regulating cellular reactive oxygen species, mitochondrial DNA damage and cell apoptosis (Sun et al., 2019b). In the bleomycin-induced lung fibrosis model, Xie et al. (2011) reported that the potential target genes of let-7f may contribute to the complex transcriptional program of pulmonary fibrosis. Herein, we continuously select let-7f-5p to explore the underlying molecular mechanism of BIPF. To the best of our knowledge, this is the first demonstration of circulating miRNA change in any animal suffering from BIPF.

We know that miRNA play important roles in the regulation of target gene expression, which are often involved in some important pathway. In this study, the pathway-enriched analysis indicated that the target genes of the DEmiRNAs were highly associated with functions in the ECM-receptor interaction and focal adhesion pathways. In the pulmonary fibrosis patients, proteomic analysis of lung tissue showed that the altered proteins mainly belonged to ECM-receptor interaction and focal adhesion pathways (Tian et al., 2019). The abnormal ECM receptor interaction and focal adhesion pathways could lead to the accumulation of ECM, which has been identified as a major driving force for the development and persistence of fibrosis diseases (Liu et al., 2021). In addition, the target gene of let-7f-5p, including *PIK3CA* gene, was determined to be key gene that was reported to play an important role in the focal adhesion pathway (Margaria et al., 2022). It is worth noting that the *PIK3CA* gene is a key component in the PI3K/Akt pathway, which plays a critical role in the development pulmonary fibrosis (Hsu et al., 2017). With the in depth study of the PI3K/Akt pathway, studies reported that the activation of some factors, such as mammalian target of rapamycin, vascular endothelial growth factor and reactive oxygen species and COX2 in the downstream of the PI3K/AKT signaling pathway can participate in pulmonary fibrosis (Laddha & Kulkarni, 2019; Wu et al., 2018b). In the BIPF model, we found that the FMD pathogen promotes PI3K/AKT/COX2 signaling pathway related genes mRNA expression in rat lung. Recently, several studies have revealed that COX2 was highly induced by many different pathogens involved in pulmonary fibrosis, and COX2 can take part in the pathological process of alveolar inflammation and pulmonary fibrosis by inducing prostaglandin synthesis and microvascular hyperplasia (Wu et al., 2018b; Li, 2009). In pulmonary fibrosis model, Moore et al. (2000) found that compared with control mice, the expression level of COX2 was increased in the blood and lung of mice at 14 and 21 d after treated with bleomycin. Our results suggest that the PI3K/AKT/COX2 signaling pathway may contribute importantly to the pathogenesis of BIPF.

**Figure 7:**
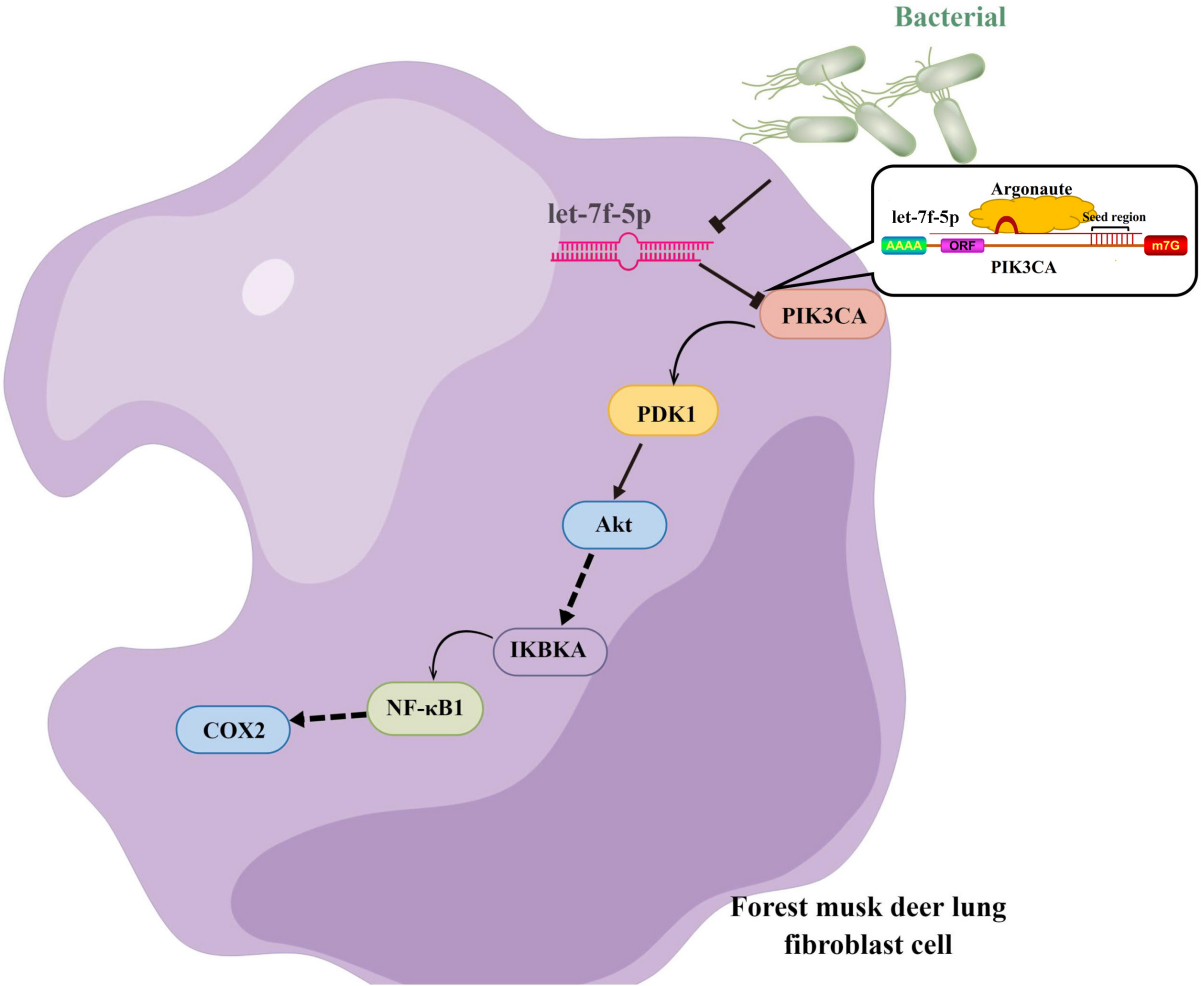
Model of the let-7f-5p regulates the PI3K/AKT/COX2 signaling pathway in forest musk deer lung fibroblast cell.

Taken together, we hypothesized that let-7f-5p is a key regulator of BIPF in FMD via targeting of the PI3K/AKT/COX2 signaling pathway. Therefore, we further demonstrated the targeting relationship between let-7f-5p and PIK3CA using bioinformatics analysis and experimental verification in HEK293T and FMD-C1 cells. This is consistent with previous research (Gilles & Slack, 2018), which showed that both let-7f-5p and its seed sequence were conserved between different species. In addition, we successfully isolated fibroblast cells from the lung of FMD, this is the first reported of fibroblast cells from FMD. As a key cell type driving the fibrogenic process, fibroblast cells can trigger pulmonary fibrosis directly through abnormal proliferating and transforming, and promote the secretion of ECM, cytokine and inflammatory cell. Moreover, various fields of pulmonary fibrosis researches have retained in relation to fibroblast cells (Wu, Tang & Kapoor, 2021). Notably, we found that let-7f-5p is a negative regulator for the PI3K/AKT/COX2 signaling pathway in FMD-C1, confirming our hypothesis that let-7f-5p could regulate PI3K/AKT/COX2 signaling pathway in BIPF through suppression of *PIK3CA* expression (Fig. 7). It is worth noting that the activation of PI3K is directly contribute to collagen- and fibro-proliferative has been confirmed repeatedly in many laboratories. Hsu et al. (2017) reported that many PI3K inhibitors have been developed for the treatment of pulmonary fibrosis. Thus, as a PI3K inhibitor, let-7f-5p may be a potential therapeutic molecule for BIPF.

## Conclusions

In conclusion, we successfully identified let-7f-5p related to BIPF in FMD by combining the analysis of circulating miRNA, establishment of BIPF rat model and culture of FMD lung fibroblast cells. Of note, let-7f-5p could regulate the PI3K/AKT/COX2 signaling pathway in BIPF through suppression of PIK3CA expression. In this article, we believe that our study particularly strengthens the design for the non-invasive research in pulmonary disease in rare animals, and also provides further insights into the molecular mechanisms of BIPF.

##  Supplemental Information

10.7717/peerj.14097/supp-1 Supplemental Information 1 Supplemental Information Table S1: RT-qPCR primers used for the verification of miRNAs; Table S2: RT-qPCR primers used for the verification of mRNAs; Table S3: Information of PCR primers for recombinant double luciferase reporter plasmids; Table S4: Overview of small RNA sequencing data in this study; Figure S1: Package of the recombinant adeno-associated virus; Figure S2: Isolation and identification of pathogens in forest musk deer lung; Figure S3: Verification of recombinant luciferase reporter plasmid.

10.7717/peerj.14097/supp-2 Data S1 Raw data

10.7717/peerj.14097/supp-3 Supplemental Information 3 Original_Images for Blots/Gels

10.7717/peerj.14097/supp-4 Supplemental Information 4 Author Check

10.7717/peerj.14097/supp-5 Supplemental Information 5 Explanation of miRNA-seq raw data

## Additional Information and Declarations

### Data Availability

The following information was supplied regarding data availability: The miRNA-sequencing raw data of forest musk deer is available at NCBI Sequence Read Archive: SRX9254510–SRX9254519.
