# Supplementary material for: MicroRNA let-7f-5p regulates PI3K/AKT/COX2 signaling pathway in bacteria-induced pulmonary fibrosis via targeting of PIK3CA in forest musk deer
Source: PeerJ. 2022 Oct 5;10:e14097. doi: 10.7717/peerj.14097 (PMC9547585; doi:10.7717/peerj.14097)
Supplement: Supplemental Information 1 — Table S1: RT-qPCR primers used for the verification of miRNAs; Table S2: RT-qPCR primers used for the verification of mRNAs; Table S3: Information of PCR primers for recombinant double luciferase reporter plasmids; Table S4: Overview of small RNA sequencing data in this study; Figure S1: Package of the recombinant adeno-associated virus; Figure S2: Isolation and identification of pathogens in forest musk deer lung; Figure S3: Verification of recombinant luciferase reporter plasmid. [file peerj-10-14097-s001.zip › Supplementary materials/Figure S1.pptx]

## Slide 1
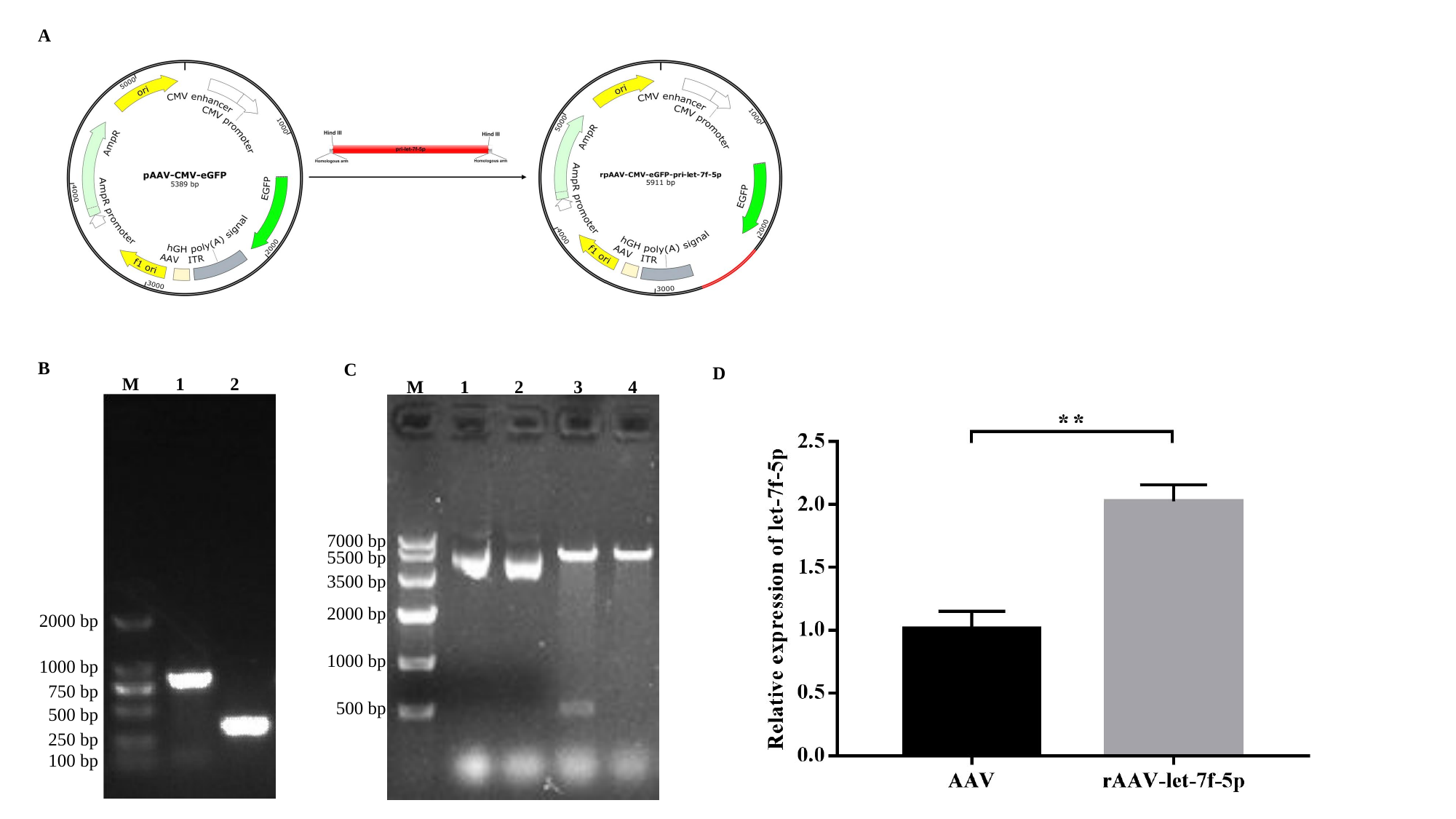

A
B
C
D
 M 1 2
2000 bp
1000 bp
750 bp
500 bp
250 bp
100 bp
 M 1 2 3 4
7000 bp
5500 bp
3500 bp
2000 bp
1000 bp
500 bp

## Slide 2
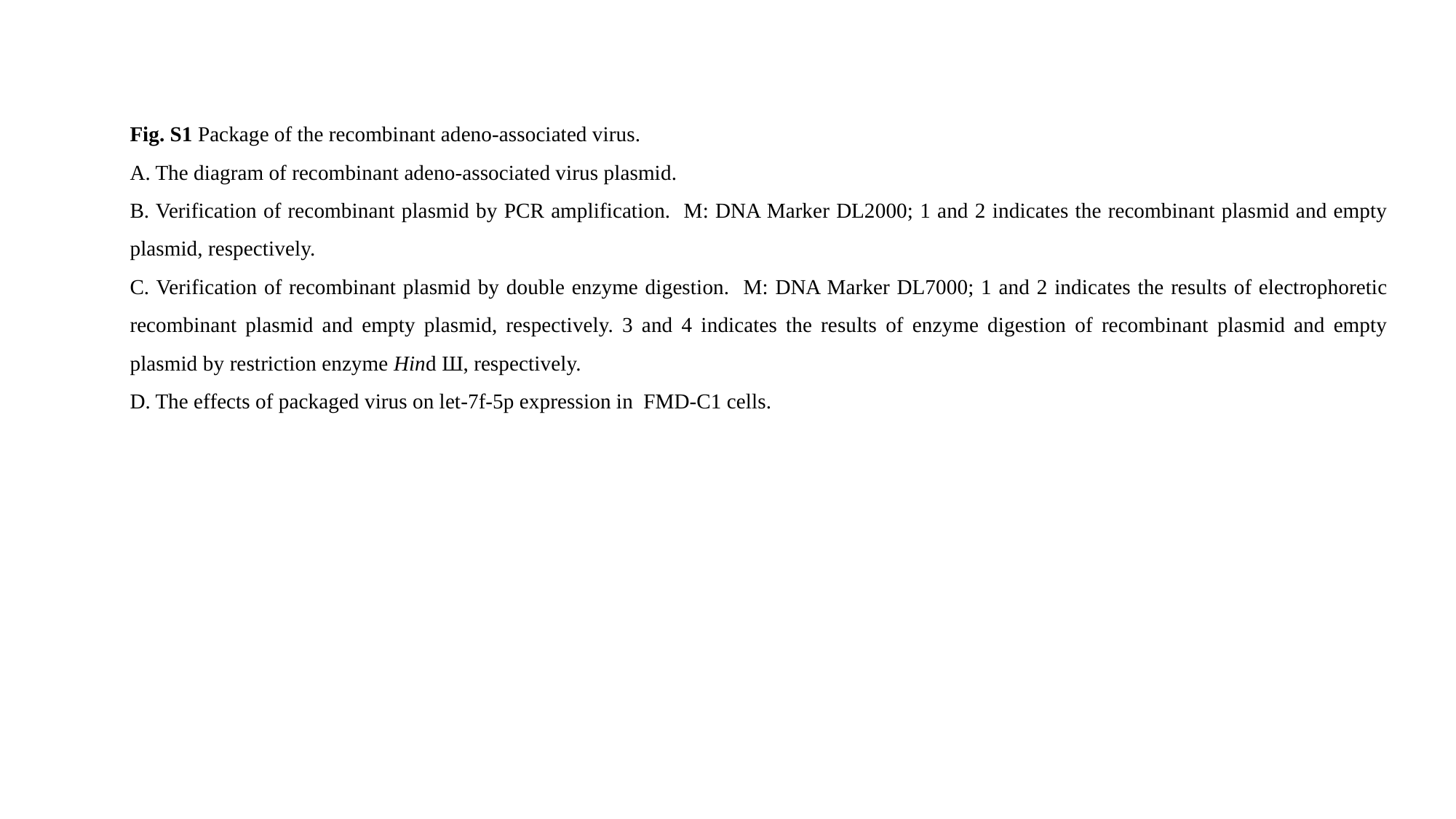

Fig. S1 Package of the recombinant adeno-associated virus.
A. The diagram of recombinant adeno-associated virus plasmid.
B. Verification of recombinant plasmid by PCR amplification. M: DNA Marker DL2000; 1 and 2 indicates the recombinant plasmid and empty plasmid, respectively.
C. Verification of recombinant plasmid by double enzyme digestion. M: DNA Marker DL7000; 1 and 2 indicates the results of electrophoretic recombinant plasmid and empty plasmid, respectively. 3 and 4 indicates the results of enzyme digestion of recombinant plasmid and empty plasmid by restriction enzyme Hind Ш, respectively.
D. The effects of packaged virus on let-7f-5p expression in FMD-C1 cells.
