# Supplementary material for: MicroRNA let-7f-5p regulates PI3K/AKT/COX2 signaling pathway in bacteria-induced pulmonary fibrosis via targeting of PIK3CA in forest musk deer
Source: PeerJ. 2022 Oct 5;10:e14097. doi: 10.7717/peerj.14097 (PMC9547585; doi:10.7717/peerj.14097)
Supplement: Supplemental Information 1 — Table S1: RT-qPCR primers used for the verification of miRNAs; Table S2: RT-qPCR primers used for the verification of mRNAs; Table S3: Information of PCR primers for recombinant double luciferase reporter plasmids; Table S4: Overview of small RNA sequencing data in this study; Figure S1: Package of the recombinant adeno-associated virus; Figure S2: Isolation and identification of pathogens in forest musk deer lung; Figure S3: Verification of recombinant luciferase reporter plasmid. [file peerj-10-14097-s001.zip › Supplementary materials/Figure S2.pptx]

## Slide 1
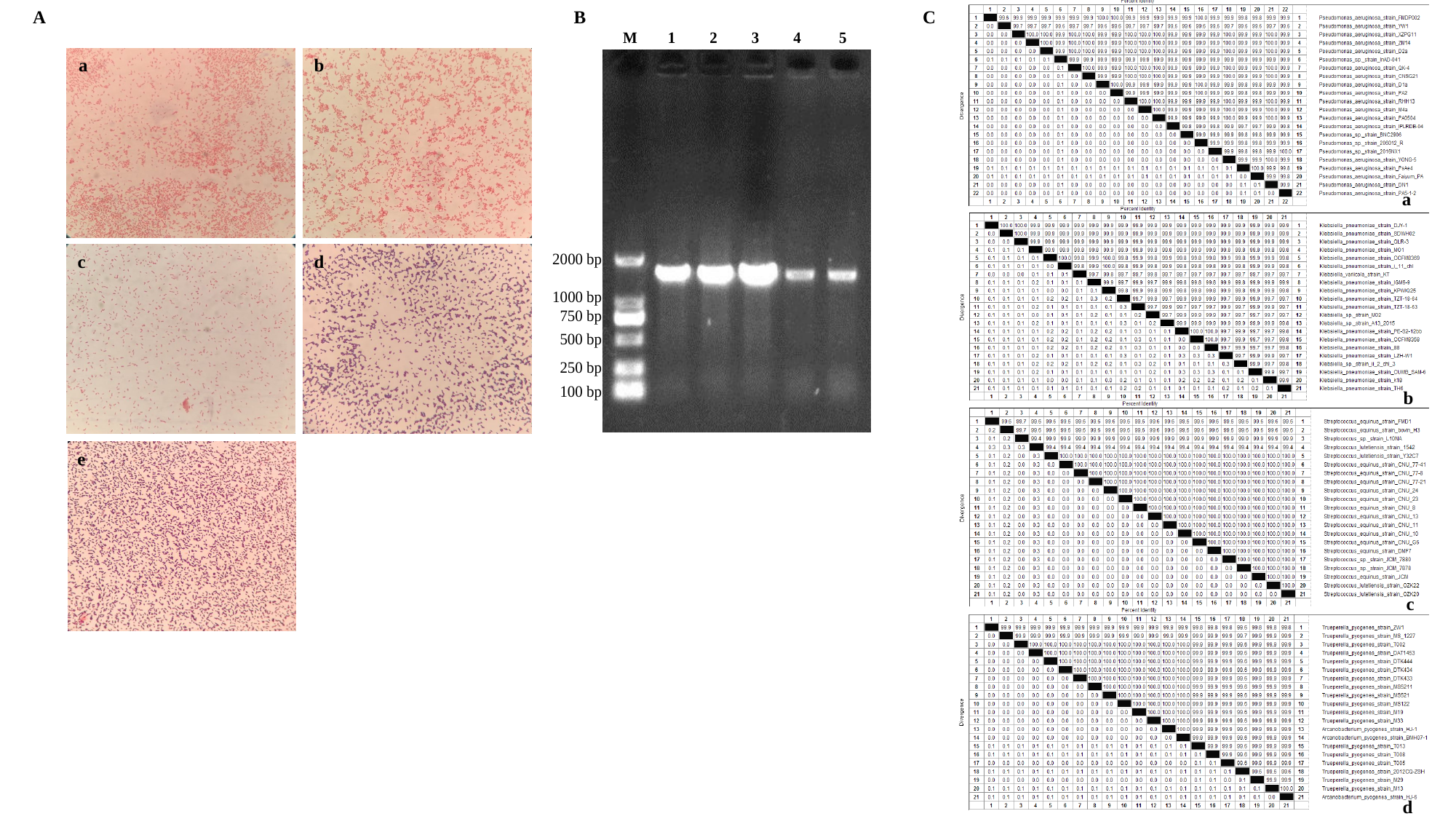

A
B
C
a
b
a
c
d
b
e
c
d

## Slide 2
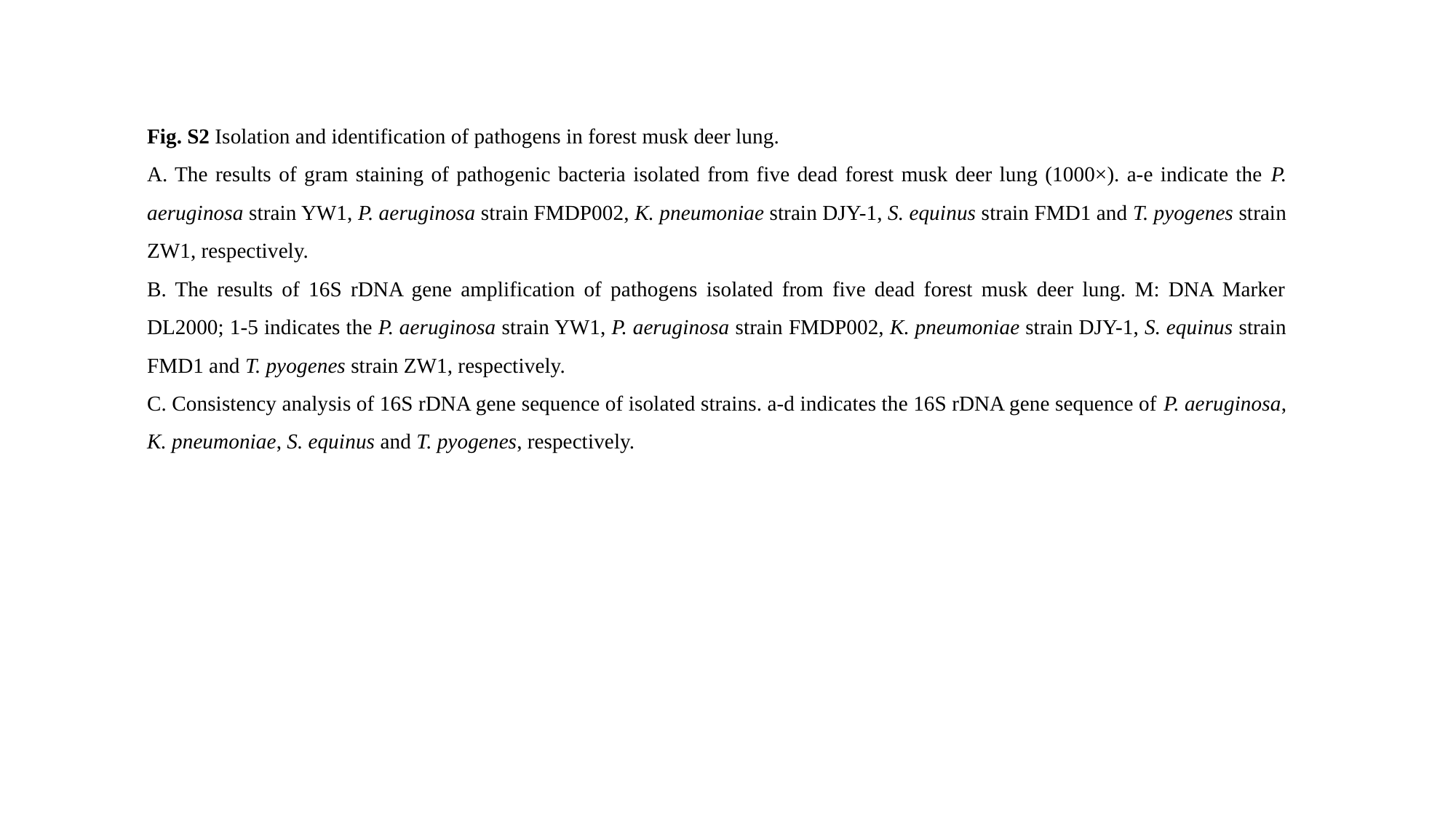

Fig. S2 Isolation and identification of pathogens in forest musk deer lung.
A. The results of gram staining of pathogenic bacteria isolated from five dead forest musk deer lung (1000×). a-e indicate the P. aeruginosa strain YW1, P. aeruginosa strain FMDP002, K. pneumoniae strain DJY-1, S. equinus strain FMD1 and T. pyogenes strain ZW1, respectively.
B. The results of 16S rDNA gene amplification of pathogens isolated from five dead forest musk deer lung. M: DNA Marker DL2000; 1-5 indicates the P. aeruginosa strain YW1, P. aeruginosa strain FMDP002, K. pneumoniae strain DJY-1, S. equinus strain FMD1 and T. pyogenes strain ZW1, respectively.
C. Consistency analysis of 16S rDNA gene sequence of isolated strains. a-d indicates the 16S rDNA gene sequence of P. aeruginosa, K. pneumoniae, S. equinus and T. pyogenes, respectively.
