# Supplementary material for: MicroRNA let-7f-5p regulates PI3K/AKT/COX2 signaling pathway in bacteria-induced pulmonary fibrosis via targeting of PIK3CA in forest musk deer
Source: PeerJ. 2022 Oct 5;10:e14097. doi: 10.7717/peerj.14097 (PMC9547585; doi:10.7717/peerj.14097)
Supplement: Supplemental Information 1 — Table S1: RT-qPCR primers used for the verification of miRNAs; Table S2: RT-qPCR primers used for the verification of mRNAs; Table S3: Information of PCR primers for recombinant double luciferase reporter plasmids; Table S4: Overview of small RNA sequencing data in this study; Figure S1: Package of the recombinant adeno-associated virus; Figure S2: Isolation and identification of pathogens in forest musk deer lung; Figure S3: Verification of recombinant luciferase reporter plasmid. [file peerj-10-14097-s001.zip › Supplementary materials/Figure S3.pptx]

## Slide 1
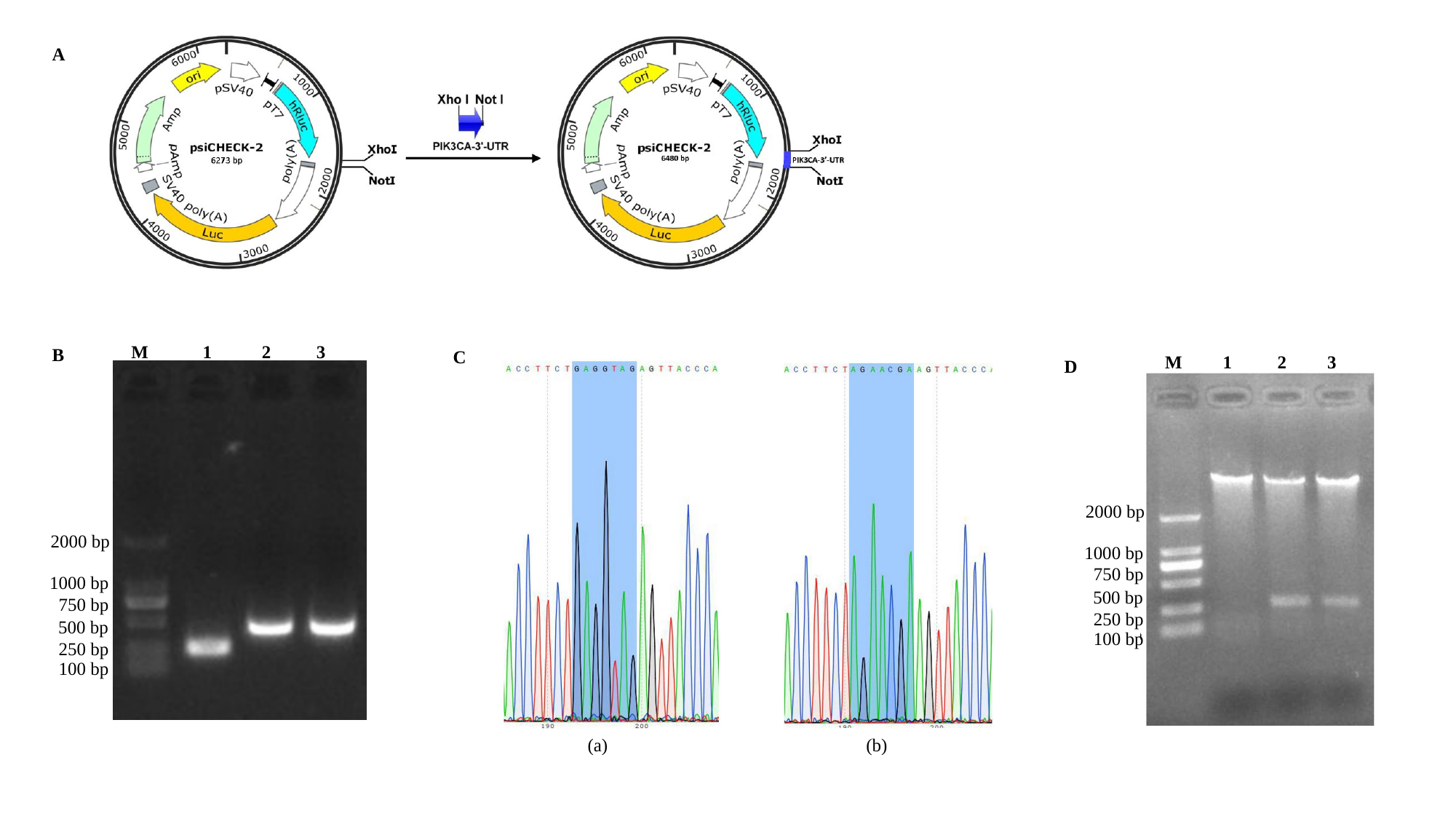

A
M 1 2 3
2000 bp
1000 bp
750 bp
250 bp
100 bp
500 bp
B
C
(a)
(b)
M 1 2 3
2000 bp
1000 bp
750 bp
250 bp
100 bp
500 bp
D

## Slide 2
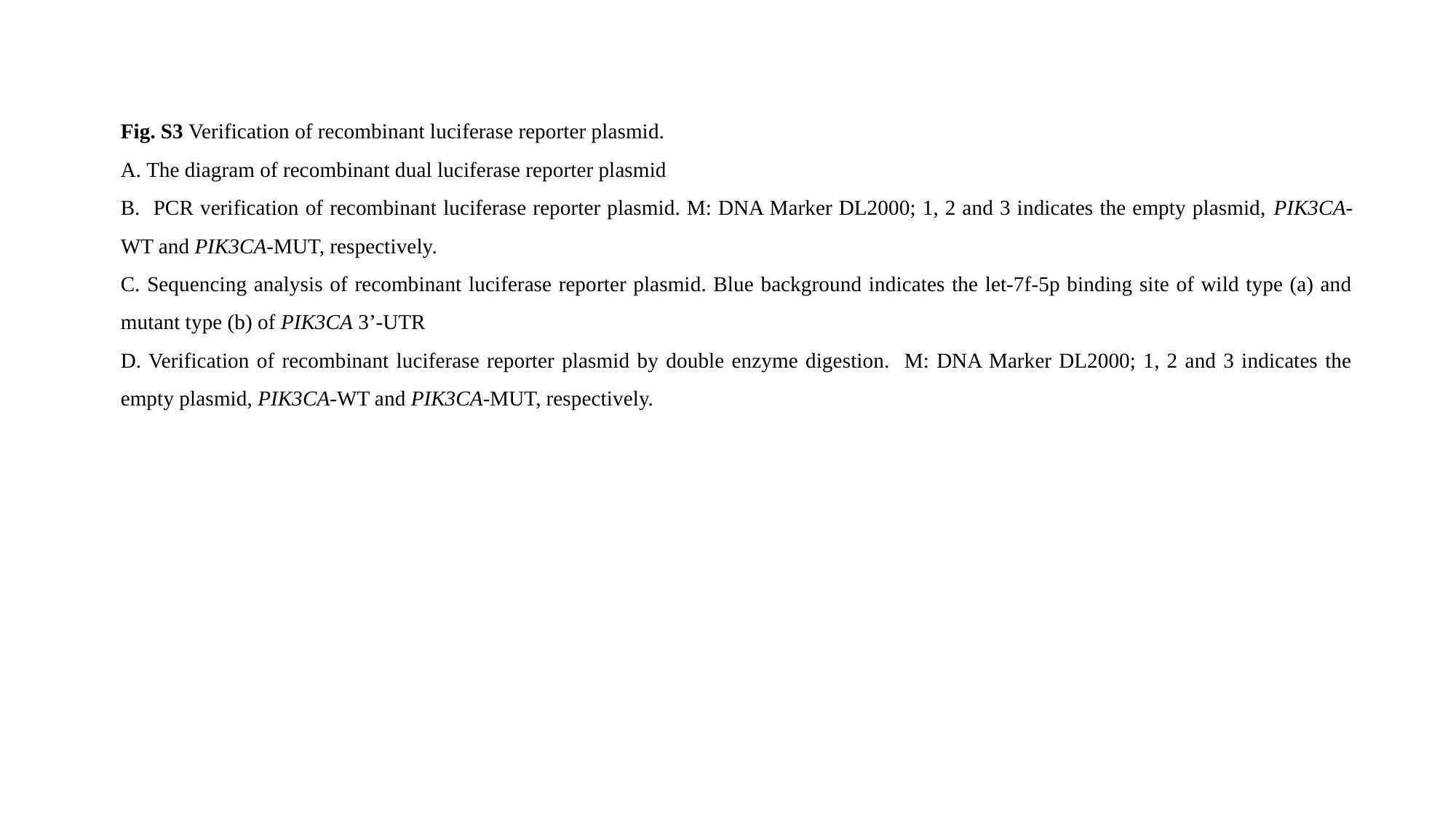

Fig. S3 Verification of recombinant luciferase reporter plasmid.
A. The diagram of recombinant dual luciferase reporter plasmid
B. PCR verification of recombinant luciferase reporter plasmid. M: DNA Marker DL2000; 1, 2 and 3 indicates the empty plasmid, PIK3CA-WT and PIK3CA-MUT, respectively.
C. Sequencing analysis of recombinant luciferase reporter plasmid. Blue background indicates the let-7f-5p binding site of wild type (a) and mutant type (b) of PIK3CA 3’-UTR
D. Verification of recombinant luciferase reporter plasmid by double enzyme digestion. M: DNA Marker DL2000; 1, 2 and 3 indicates the empty plasmid, PIK3CA-WT and PIK3CA-MUT, respectively.
