# Supplementary material for: MicroRNA let-7f-5p regulates PI3K/AKT/COX2 signaling pathway in bacteria-induced pulmonary fibrosis via targeting of PIK3CA in forest musk deer
Source: PeerJ. 2022 Oct 5;10:e14097. doi: 10.7717/peerj.14097 (PMC9547585; doi:10.7717/peerj.14097)
Supplement: Supplemental Information 1 — Table S1: RT-qPCR primers used for the verification of miRNAs; Table S2: RT-qPCR primers used for the verification of mRNAs; Table S3: Information of PCR primers for recombinant double luciferase reporter plasmids; Table S4: Overview of small RNA sequencing data in this study; Figure S1: Package of the recombinant adeno-associated virus; Figure S2: Isolation and identification of pathogens in forest musk deer lung; Figure S3: Verification of recombinant luciferase reporter plasmid. [file peerj-10-14097-s001.zip › Supplementary materials/Table S1.docx]

**Table S1.** RT-qPCR primers used for the verification of miRNAs

| Target gene | Forward (5’→3’) | Reverse (5’→3’) |
| --- | --- | --- |
| miR-30 | GCTGTAAACATCCTTCACTCTCAGC | - |
| let-7f-5p | GCCGGTGAGGTAGTAGATTGTATAGTT | - |
| miR-27d-3p | GGTTCACAGTGGCTAAGTTCGG | - |
| miR-25-3p | GCATTGCACTTGTCTCGGC | - |
| miR-142-5p | GGCCCATAAAGTAGAAAGCACTAC | - |
| miR-451-3p | GCATGGTAACGGTTCTCTTGCTG | - |
| miR-652 | TGAATGGCGCCACTAGGGTTGT | - |
| miR-9-5p | GCGTCTTTGGTTATCTAGCTGTGTG | - |
| miR-206 | GCTGGAATGTAAGGAAGTGTGTGG | - |
| *cel*-miR-39 | TCACCGGGTGTAAATCAGCTTG | - |
| U6 | CTCGCTTCGGCAGCACA | AACGCTTCACGAATTTGCGT |

“-” indicates the reverse primer was provided in miRcute Plus miRNA qPCR Kit (SYBR Green) (TianGen, Beijing, China).
