# Supplementary material for: MicroRNA let-7f-5p regulates PI3K/AKT/COX2 signaling pathway in bacteria-induced pulmonary fibrosis via targeting of PIK3CA in forest musk deer
Source: PeerJ. 2022 Oct 5;10:e14097. doi: 10.7717/peerj.14097 (PMC9547585; doi:10.7717/peerj.14097)
Supplement: Supplemental Information 1 — Table S1: RT-qPCR primers used for the verification of miRNAs; Table S2: RT-qPCR primers used for the verification of mRNAs; Table S3: Information of PCR primers for recombinant double luciferase reporter plasmids; Table S4: Overview of small RNA sequencing data in this study; Figure S1: Package of the recombinant adeno-associated virus; Figure S2: Isolation and identification of pathogens in forest musk deer lung; Figure S3: Verification of recombinant luciferase reporter plasmid. [file peerj-10-14097-s001.zip › Supplementary materials/Table S2.docx]

**Table S2** RT-qPCR primers used for the verification of mRNAs

| Gene | GenBank NO. | Sequence (5’→3’)^1^ | Tm (℃) | Product size (bp) | Host |
| --- | --- | --- | --- | --- | --- |
| *TGF-β1* | AY550025.1 | F: GAGGAGCAGGAAGGGTC  R: AGGAGACGGAATACAGGG | 58 | 151 | Rat |
| *TNF-α* | HQ201305.1 | F: CACCACGCTCTTCTGTCT  R: ACGGGCTTGTCACTCG | 58 | 144 | Rat |
| *PIK3CA* | XM_017602181.1 | F: CTCCGTGAGGCCACACTA  R: ATTCTTCCCTTTCTGCTT | 52 | 136 | Rat |
| *PDK1* | NM_031081.1 | F: GTTGCCTCCAAACCTCC  R: TCACAAGAACTCCGACCAG | 61 | 136 | Rat |
| *Akt1* | XM_032894102.1 | F: GGCTGGGCGTGGTCATGT  R: CTCTGCTTAGGGTCCTTC | 52 | 172 | Rat |
| *IKBKA* | NM_001107588.1 | F: GATGTTCACGGTCTGC  R: CCCAAGGTGGAAGTG | 52 | 211 | Rat |
| *NF-KB1* | XM_032897276.1 | F: CTTCTTTGGCAGCTAGGTG  R: TGGAGGACTTGCTGAGGGT | 60 | 84 | Rat |
| *COX2* | NM_017232.3 | F: TCGGAGGAGAAGTGGGTT  R: GAAAGAGGCAAAGGGACA | 52 | 92 | Rat |
| *β-actin* | XM_032903898.1 | F: GAATCTGCTGGCATTCACG  R: TCTTCATGGTGCTGGGAGC | 58 | 172 | Rat |
| *PIK3CA* | SPDX01000374.1 | F: CTCCGTGAGGCTACATTA  R: ATTCTTCCCTTTCTGCTT | 58 | 136 | Forest musk deer |
| *Akt1* | SPDX01013471.1 | F: GGCTGGGCGTGGTCATGT  R: CGCTGCTTGGGGTCCTTC | 58 | 131 | Forest musk deer |
| *PDK1* | SPDX01009946.1 | F: TTTCTTCCGAGGCTGTGGG  R: GCGTGGTGTTATGCTCCTGC | 58 | 185 | Forest musk deer |
| *IKBKA* | SPDX01010198.1 | F: GTTTCCCGCTCAATAC  R: ACTTCGGCAAAGATAAT | 50 | 80 | Forest musk deer |
| *NF-KB1* | SPDX01002780.1 | F: CTTCTTTGGCAGCGAGGTG  R: TGGAGGACTTGCTGAGGGC | 60 | 84 | Forest musk deer |
| *COX2* | SPDX01007628.1 | F: TTGGTGGAGAAGTAGGTT  R: GAAAGAGGTAAAGGGACA | 52 | 92 | Forest musk deer |
| *β-actin* | SPDX01033109.1 | F: GAATCCTGCGGCATTCACG  R: TCTTCATCGTGCTGGGTGC | 58 | 172 | Forest musk deer |

^1^“F” indicates the forward primer; “R” indicates the reverse primer
