# Supplementary material for: MicroRNA let-7f-5p regulates PI3K/AKT/COX2 signaling pathway in bacteria-induced pulmonary fibrosis via targeting of PIK3CA in forest musk deer
Source: PeerJ. 2022 Oct 5;10:e14097. doi: 10.7717/peerj.14097 (PMC9547585; doi:10.7717/peerj.14097)
Supplement: Supplemental Information 1 — Table S1: RT-qPCR primers used for the verification of miRNAs; Table S2: RT-qPCR primers used for the verification of mRNAs; Table S3: Information of PCR primers for recombinant double luciferase reporter plasmids; Table S4: Overview of small RNA sequencing data in this study; Figure S1: Package of the recombinant adeno-associated virus; Figure S2: Isolation and identification of pathogens in forest musk deer lung; Figure S3: Verification of recombinant luciferase reporter plasmid. [file peerj-10-14097-s001.zip › Supplementary materials/Table S3..docx]

**Table S3** Information of PCR primers for recombinant double luciferase reporter plasmids

| Template | Sequence (5’ →3’)^1^ | Tm (℃) | Product size (bp) |
| --- | --- | --- | --- |
| psiCHECK^TM^-2 vector | F: CGTCAGACAAACCCTAACCA  R: CACTTCGCCAGGAGGAC | 55 | 195 |
| PIK3CA-WT vector |  |  | 402 |
| PIK3CA-MUT vector |  |  | 402 |

^1^“F” indicates the forward primer; “R” indicates the reverse primer
