# Supplementary material for: MicroRNA let-7f-5p regulates PI3K/AKT/COX2 signaling pathway in bacteria-induced pulmonary fibrosis via targeting of PIK3CA in forest musk deer
Source: PeerJ. 2022 Oct 5;10:e14097. doi: 10.7717/peerj.14097 (PMC9547585; doi:10.7717/peerj.14097)
Supplement: Supplemental Information 1 — Table S1: RT-qPCR primers used for the verification of miRNAs; Table S2: RT-qPCR primers used for the verification of mRNAs; Table S3: Information of PCR primers for recombinant double luciferase reporter plasmids; Table S4: Overview of small RNA sequencing data in this study; Figure S1: Package of the recombinant adeno-associated virus; Figure S2: Isolation and identification of pathogens in forest musk deer lung; Figure S3: Verification of recombinant luciferase reporter plasmid. [file peerj-10-14097-s001.zip › Supplementary materials/Table S4.docx]

**Table S4** Overview of small RNA sequencing data in this study.

| Sample^1^ | Raw data Reads | Clean data Reads (≥18 nt) | Clean ratio |
| --- | --- | --- | --- |
| H1 | 38431176 | 37027875 | 96.35% |
| H2 | 25061456 | 23462618 | 93.62% |
| H3 | 40568382 | 38548693 | 95.02% |
| H4 | 36374798 | 31910174 | 87.73% |
| H5 | 26773697 | 26177644 | 97.77% |
| P1 | 31489733 | 29105578 | 92.43% |
| P2 | 37659898 | 35103951 | 93.21% |
| P3 | 35860453 | 32986116 | 91.98% |
| P4 | 42634692 | 27518271 | 64.54% |
| P5 | 41644307 | 31510889 | 75.67% |

^1^ H and P indicates healthy and dead group, respectively.
