# Supplementary material for: MicroRNA let-7f-5p regulates PI3K/AKT/COX2 signaling pathway in bacteria-induced pulmonary fibrosis via targeting of PIK3CA in forest musk deer
Source: PeerJ. 2022 Oct 5;10:e14097. doi: 10.7717/peerj.14097 (PMC9547585; doi:10.7717/peerj.14097)
Supplement: Data S1 [file peerj-10-14097-s002.zip › Raw data/Fig.4/KEGG analysis.docx]

KEGG enrichment analysis of target genes of nine differentially expressed miRNAs

| Rank | KEGG Pathways | Target Genes | p-value | FDR^a)^ |
| --- | --- | --- | --- | --- |
| 1 | Olfactory transduction | 16 | 2.00445E-08 | 6.79508E-06 |
| 2 | Axon guidance | 88 | 7.72678E-06 | 0.001205408 |
| 3 | ABC transporters | 34 | 1.06673E-05 | 0.001205408 |
| 4 | ECM-receptor interaction | 44 | 1.71932E-05 | 0.001457125 |
| 5 | Fatty acid biosynthesis | 6 | 3.11572E-05 | 0.002112458 |
| 6 | Bile secretion | 38 | 0.000188414 | 0.010645395 |
| 7 | Focal adhesion | 91 | 0.000248674 | 0.012042906 |
| 8 | Aminoacyl-tRNA biosynthesis | 42 | 0.000420139 | 0.017283098 |
| 9 | Dilated cardiomyopathy | 51 | 0.000458843 | 0.017283098 |
| 10 | Arrhythmogenic right ventricular cardiomyopathy | 42 | 0.000566274 | 0.0191967 |
| 11 | Hypertrophic cardiomyopathy | 46 | 0.000710711 | 0.021902822 |
| 12 | Endocrine and other factor-regulated calcium reabsorption | 22 | 0.001043918 | 0.02949068 |

1. FDR indicates the false discovery rate.
