# Supplementary material for: MicroRNA let-7f-5p regulates PI3K/AKT/COX2 signaling pathway in bacteria-induced pulmonary fibrosis via targeting of PIK3CA in forest musk deer
Source: PeerJ. 2022 Oct 5;10:e14097. doi: 10.7717/peerj.14097 (PMC9547585; doi:10.7717/peerj.14097)
Supplement: Supplemental Information 3 [file peerj-10-14097-s003.pdf]

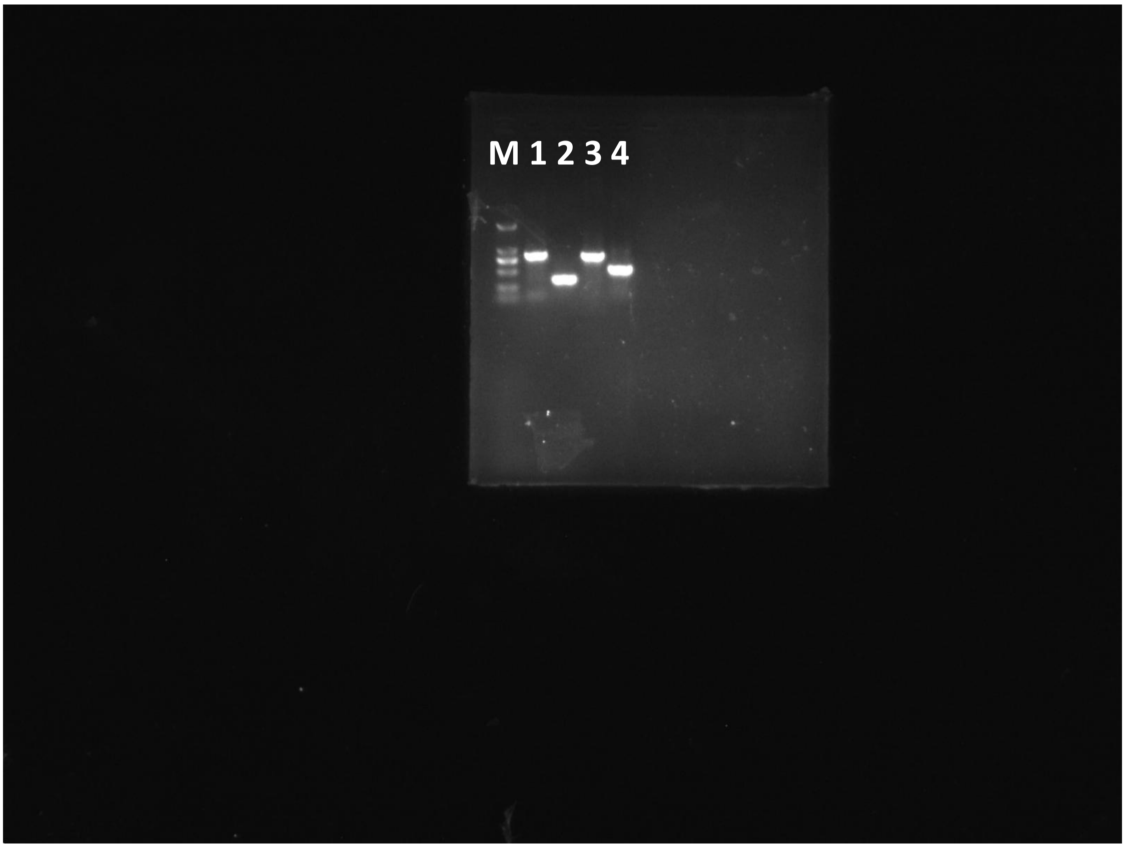

Original Image 1 (Supplementary Figure S1B): Verification of recombinant adeno-associated virus plasmid by PCR amplification. M: DNA Marker DL2000; 1-4 indicates the recombinant plasmid, empty plasmid, helper plasmid 1 and helper plasmid 2 , respectively.

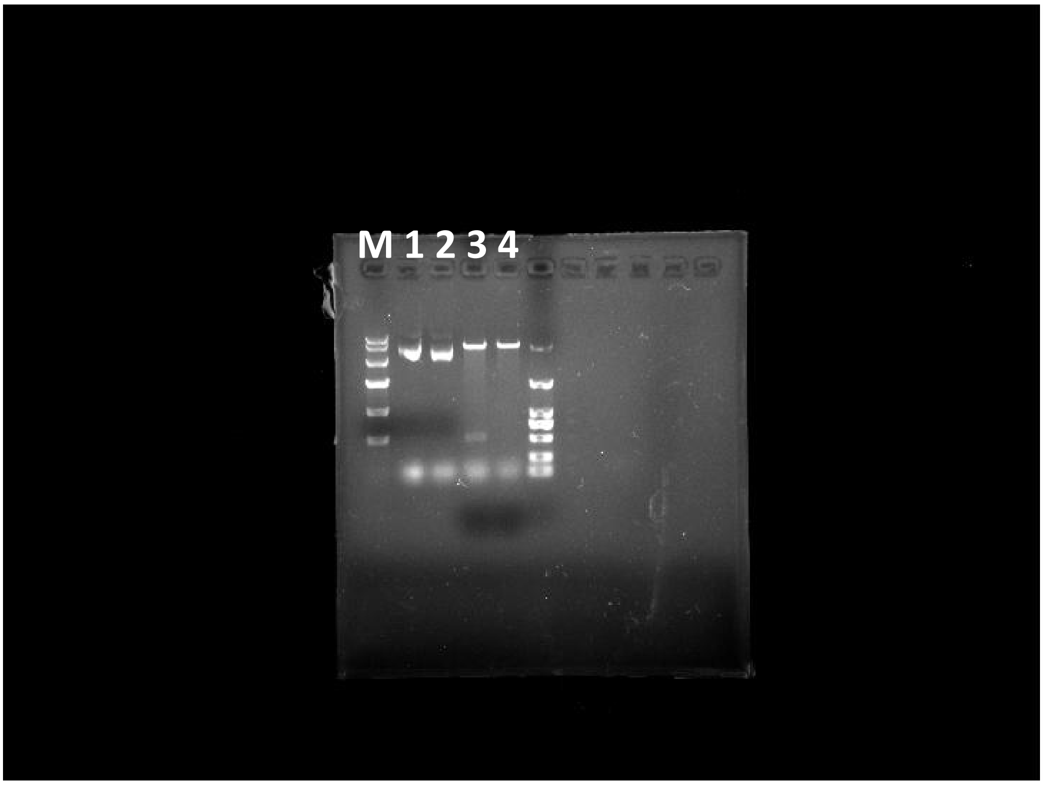

Original Image 2 (Supplementary Figure S1C): Verification of recombinant adeno-associated virus plasmid by double enzyme digestion. M: DNA Marker DL7000; 1 and 2 indicates the results of electrophoretic recombinant plasmid and empty plasmid, respectively. 3 and 4 indicates the results of enzyme digestion of recombinant plasmid and empty plasmid by restriction enzyme *Hind* Ш, respectively.

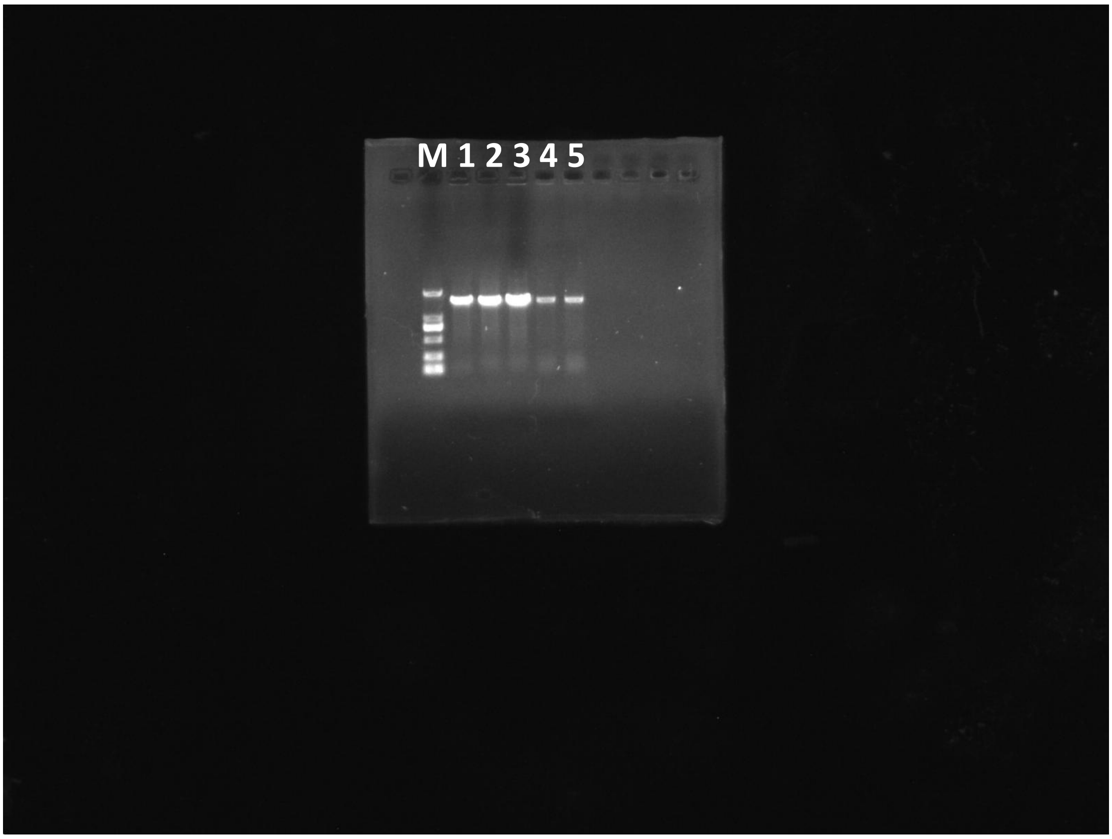

Original Image 3 (Supplementary Figure S2B): The results of 16S rDNA gene amplification of pathogens isolated from five dead forest musk deer lung. M: DNA Marker DL2000; 1-5 indicates the *P. aeruginosa* strain YW1, *P. aeruginosa* strain FMDP002, *K. pneumoniae* strain DJY-1, *S. equinus* strain FMD1 and *T. pyogenes* strain ZW1, respectively.

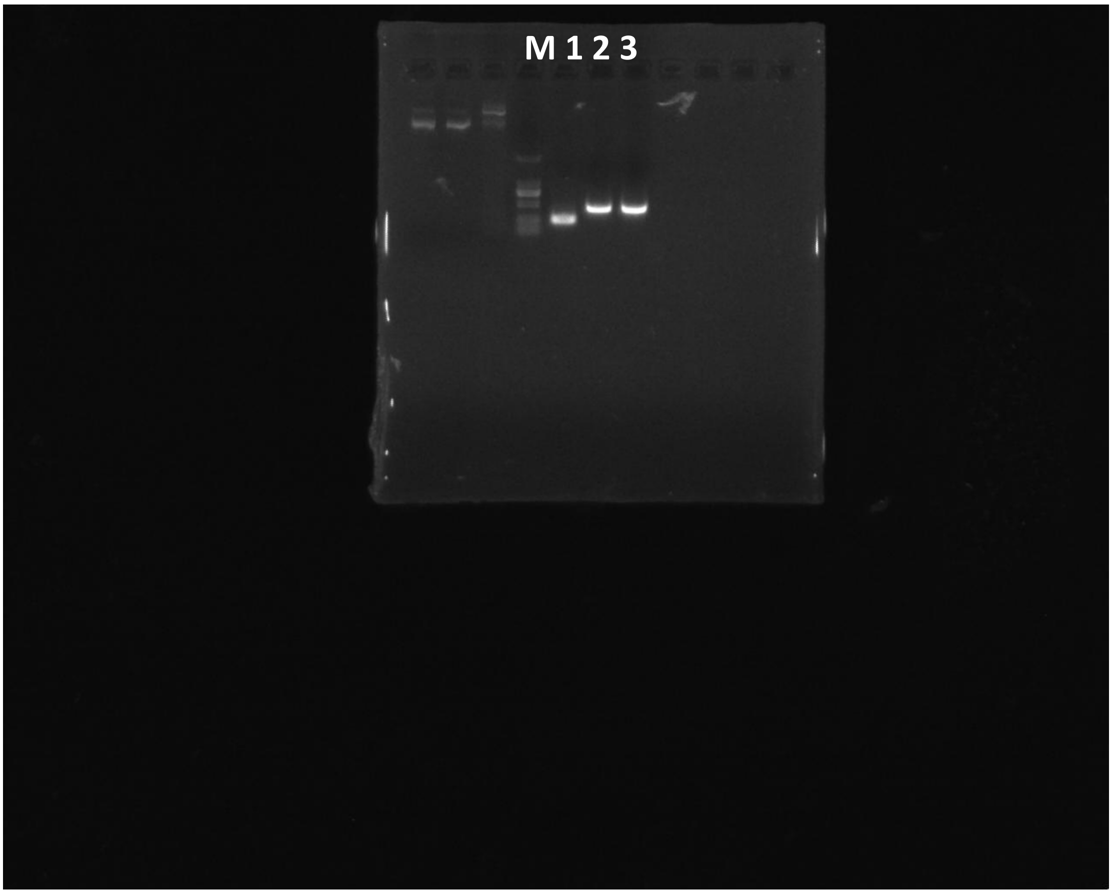

Original Image 4 (Supplementary Figure S3B): PCR verification of recombinant luciferase reporter plasmid. M: DNA Marker DL2000; 1, 2 and 3 indicates the empty plasmid, PIK3CA-WT and PIK3CA-MUT, respectively.

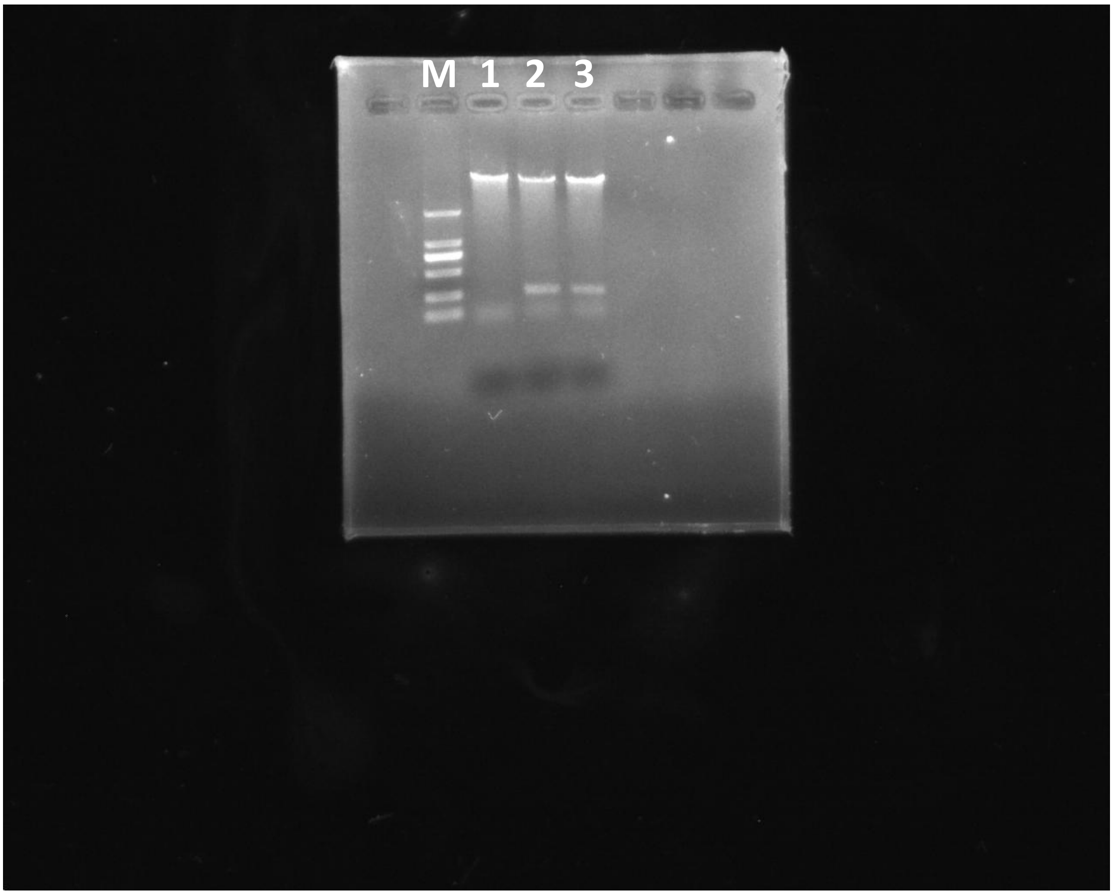

Original Image 5 (Supplementary Figure S3D): Verification of recombinant luciferase reporter plasmid by double enzyme digestion. M: DNA Marker DL2000; 1, 2 and 3 indicates the empty plasmid, PIK3CA-WT and PIK3CA-MUT, respectively.

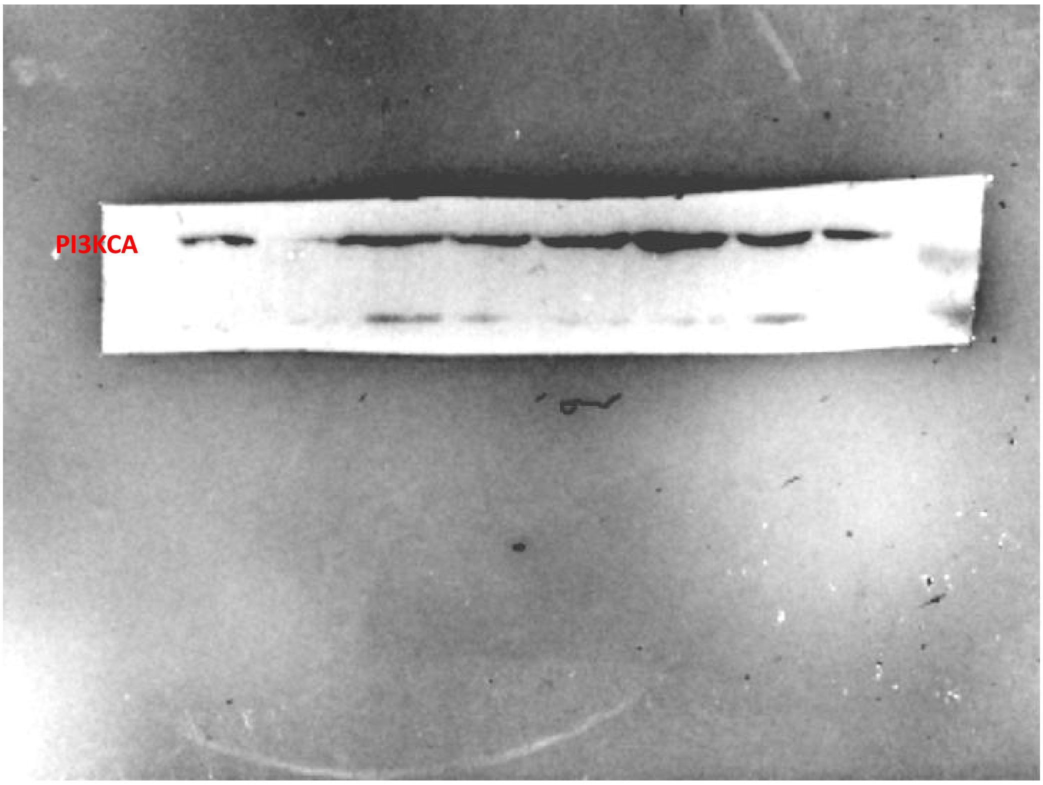

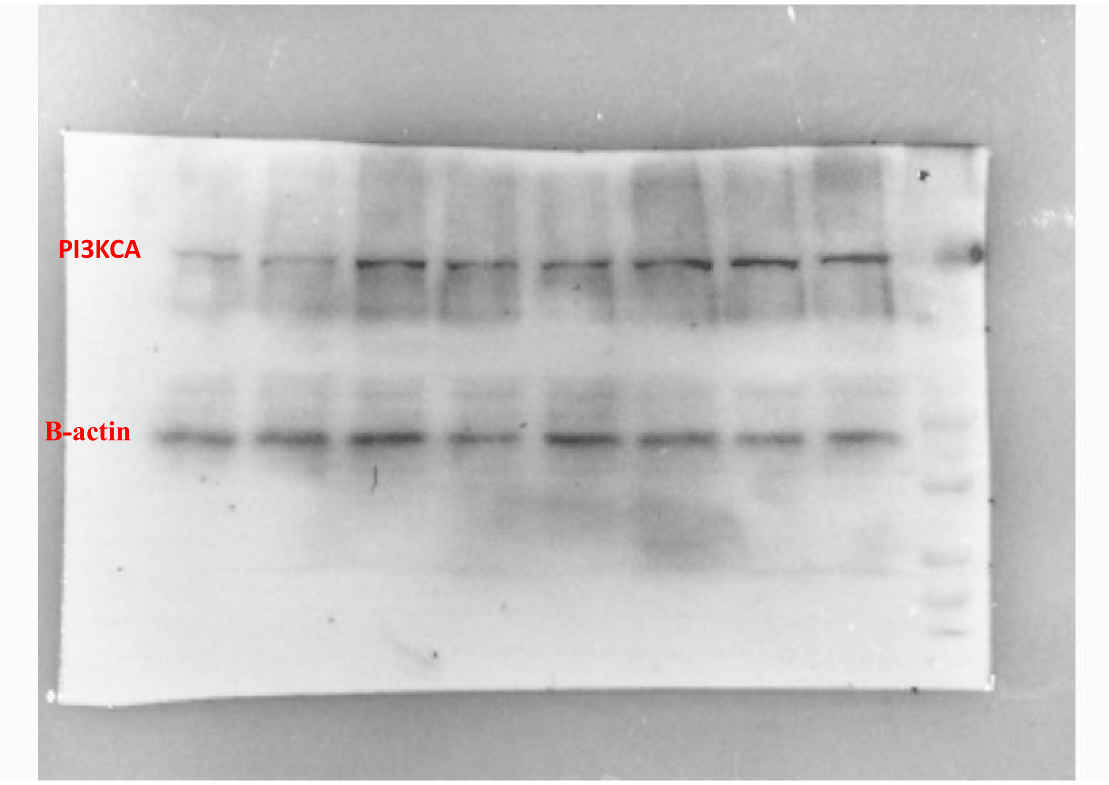

Original Image 6 (Figure 5B): Western blotting analysis for quantification of PI3KCA and β-actin proteins in four group rat lungs.

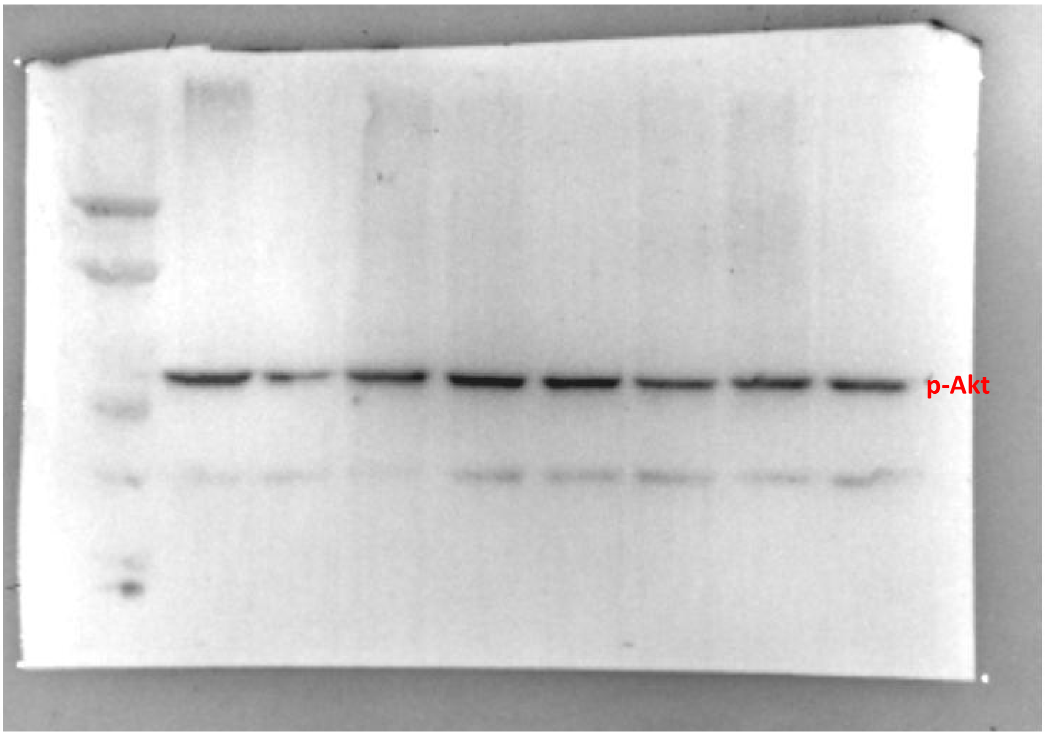

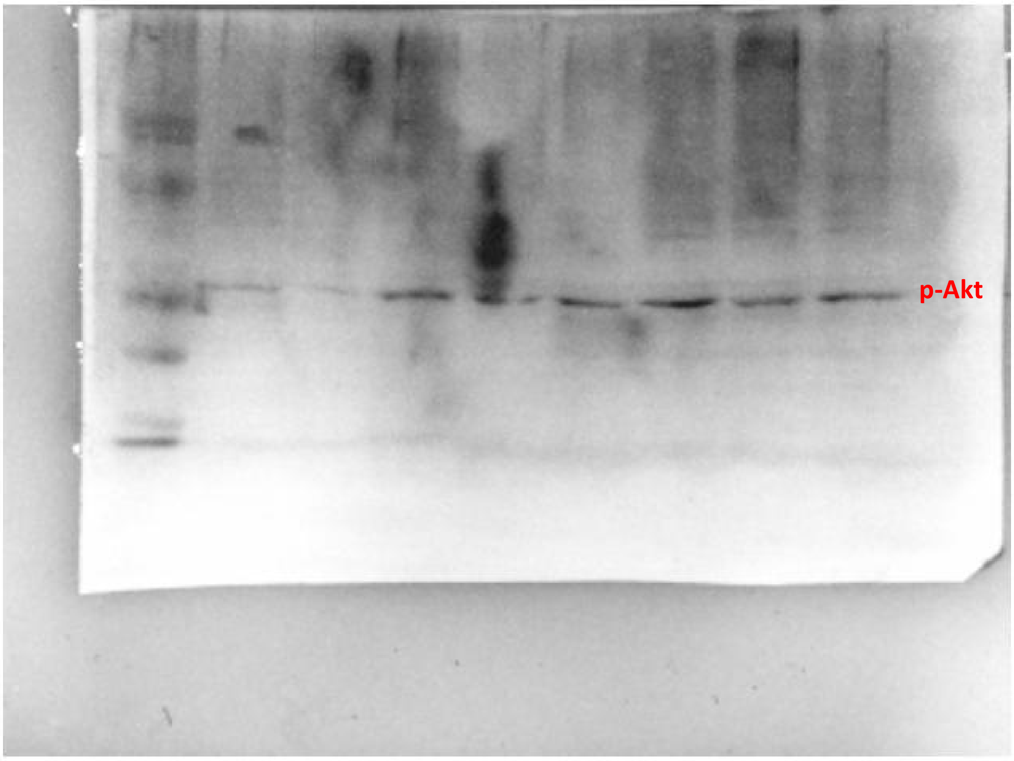

Original Image 7 (Figure 5B): Western blotting analysis for quantification of p-AKT protein in four group rat lungs.

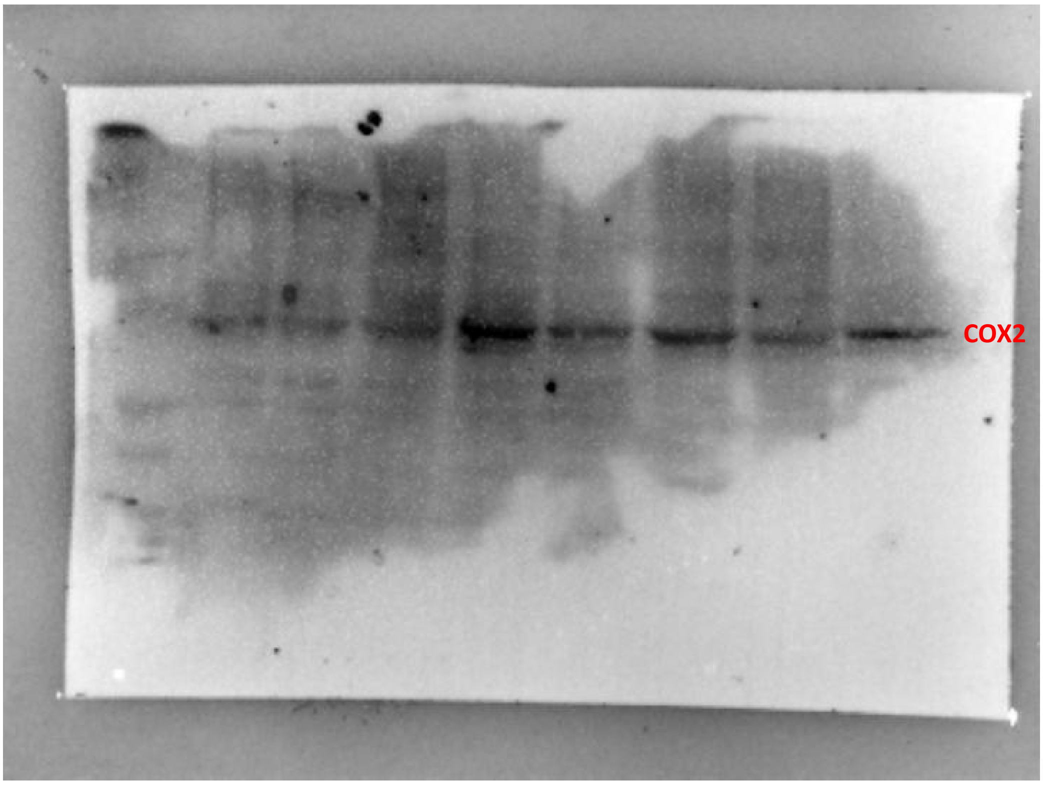

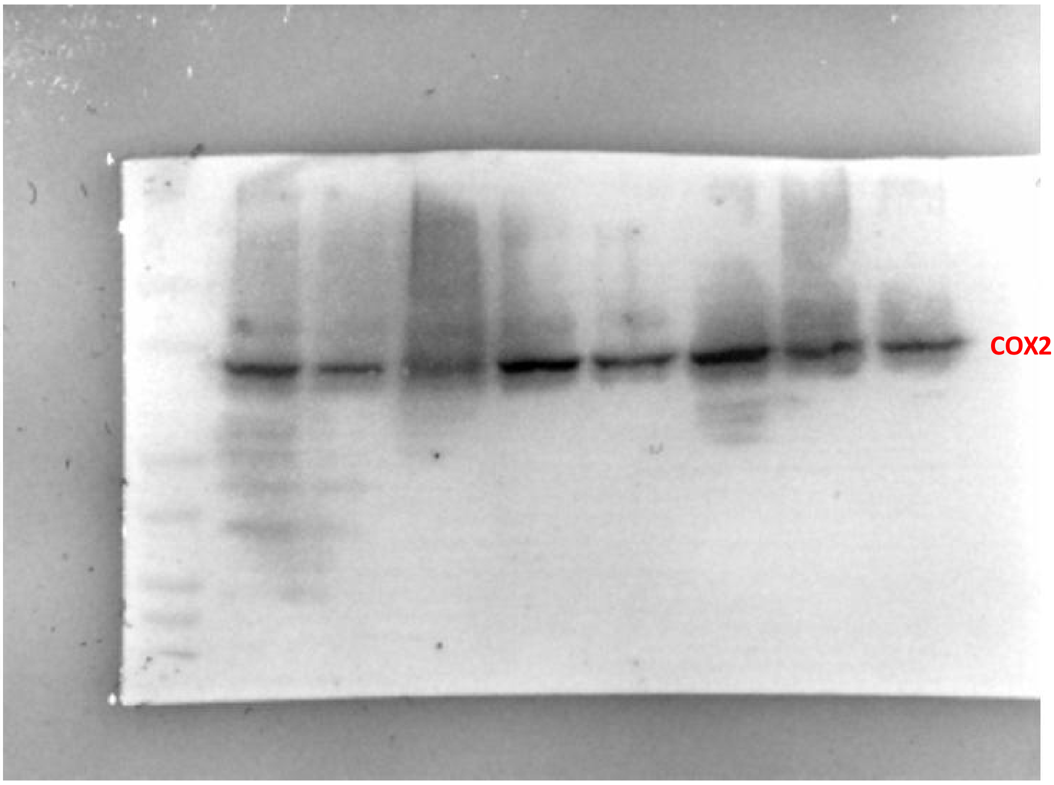

Original Image 8 (Figure 5B): Western blotting analysis for quantification of COX2 protein in four group rat lungs.

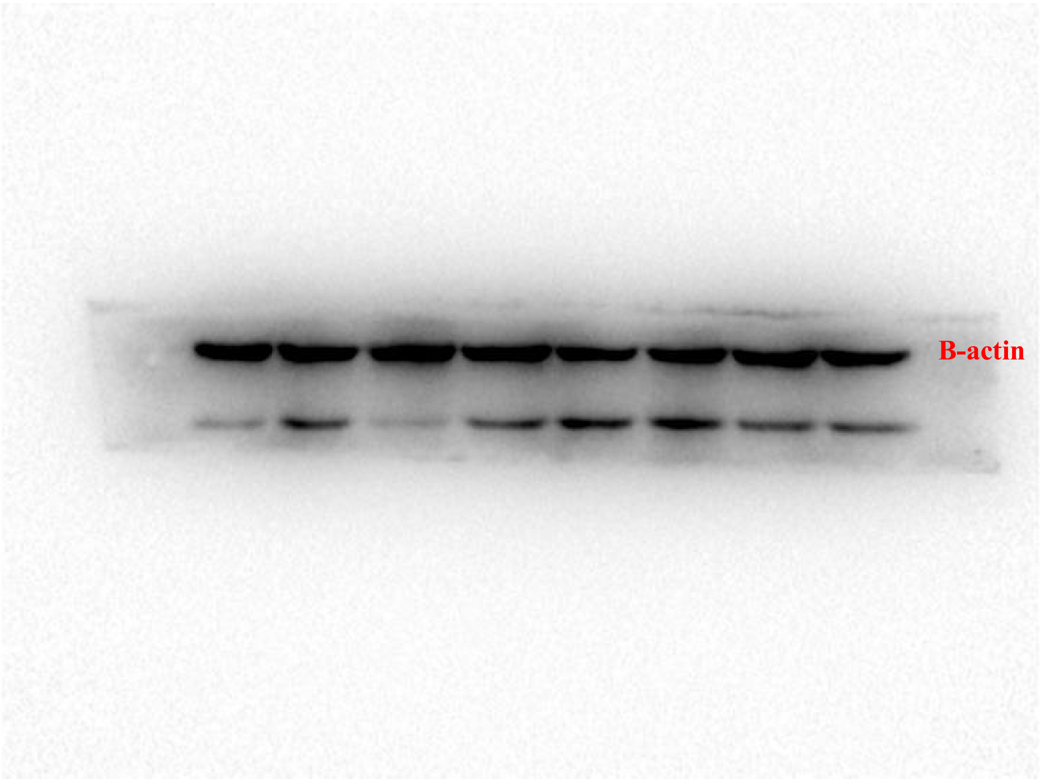

Original Image 9 (Figure 5B): Western blotting analysis for quantification of β-actin protein in four group rat lungs.
