# Supplementary material for: MicroRNA let-7f-5p regulates PI3K/AKT/COX2 signaling pathway in bacteria-induced pulmonary fibrosis via targeting of PIK3CA in forest musk deer
Source: PeerJ. 2022 Oct 5;10:e14097. doi: 10.7717/peerj.14097 (PMC9547585; doi:10.7717/peerj.14097)
Supplement: Supplemental Information 5 [file peerj-10-14097-s005.docx]

The blood miRNA-sequencing data are deposited in the NCBI Sequence Read Archive database under the accession number SRX9254510-SRX9254519.

In this study, the number of biological replicates of the miRNA-sequencing was shown in line 113 and 133. However, just like any other studies (Jie et al., 2021; Slota et al., 2019), it is failure to present the sequencing depth. Because the read levels of each miRNA were different, and the sequencing depth of current sequencing platforms exceeds that is necessary to quantify miRNAs (Vigneault et al., 2012). Besides, the raw reads and clean reads of each FMD blood sample were shown in Table S4 (> 23 million reads were sequenced for each sample).

Jie H, Xu Z, Gao J, Li F, Chen Y, Zeng D, et al. 2021. Differential expression profiles of microRNAs in musk gland of unmated and mated forest musk deer (*Moschus berezovskii*). *PeerJ*, 9:e12710. DOI:10.7717/peerj.12710.

Slota JA, Medina SJ, Klassen M, Gorski D, Mesa C.M, Robertson C, et al. 2019. Identification of circulating microRNA signatures as potential biomarkers in the serum of elk infected with chronic wasting disease. *Scientific Reports* 9:19705.DOI:10.1038/s41598-019-56249-6.

Vigneault F, Ter-Ovanesyan D, Alon S, Eminaga S, C Christodoulou D, Seidman JG, et al. 2012. High-throughput multiplex sequencing of miRNA. *Curr Protoc Hum Genet*. 11:1-10. DOI:10.1002/0471142905.hg1112s73.
